# Stromal cells downregulate *miR-23a-5p* to activate protective autophagy in acute myeloid leukemia

**DOI:** 10.1038/s41419-019-1964-8

**Published:** 2019-09-30

**Authors:** Saravanan Ganesan, Hamenth Kumar Palani, Vairavan Lakshmanan, Nithya Balasundaram, Ansu Abu Alex, Sachin David, Arvind Venkatraman, Anu Korula, Biju George, Poonkuzhali Balasubramanian, Dasaradhi Palakodeti, Neha Vyas, Vikram Mathews

**Affiliations:** 1 Department of Haematology, Christian Medical College, Vellore, India; 2 Institute for Stem Cell Biology and Regenerative Medicine (InStem), Bengaluru, India; 3 Molecular Medicine Department, St. John’s Research Institute, St. John’s National Academy of Health Sciences, Bengaluru, India

**Keywords:** Cancer microenvironment, Acute myeloid leukaemia

## Abstract

Complex molecular cross talk between stromal cells and the leukemic cells in bone marrow is known to contribute significantly towards drug-resistance. Here, we have identified the molecular events that lead to stromal cells mediated therapy-resistance in acute myeloid leukemia (AML). Our work demonstrates that stromal cells downregulate *miR-23a-5p* levels in leukemic cells to protect them from the chemotherapy induced apoptosis. Downregulation of *miR-23a-5p* in leukemic cells leads to upregulation of protective autophagy by targeting TLR2 expression. Further, autophagy inhibitors when used as adjuvants along with conventional drugs can improve drug sensitivity in vitro as well in vivo in a mouse model of leukemia. Our work also demonstrates that this mechanism of bone marrow stromal cell mediated regulation of *miR-23a-5p* levels and subsequent molecular events are relevant predominantly in myeloid leukemia. Our results illustrate the critical and dynamic role of the bone marrow microenvironment in modulating miRNA expression in leukemic cells which could contribute significantly to drug resistance and subsequent relapse, possibly through persistence of minimal residual disease in this environment.

## Introduction

Bone-marrow microenvironment is known to be actively involved in an onco-protective role for metastatic cancer cells, as well as in leukemia^1,2^. Several studies have demonstrated that tumor-stroma cross talk can play a major role in drug resistance and cancer relapse by retaining minimal residual disease^3^. The molecular mechanism and molecular players involved in contributing towards minimal residual disease and drug resistance are highly dynamic. This can be achieved by stroma-derived secretory factors such as cytokines and chemokines, which help in homing, survival, and growth of the cancer cell in bone-marrow, as well as provide protection against drug toxicity^4^. Apart from several secretory factors, microenvironment mediated changes in micro-RNA (miRNA) expression in tumor cells for cancer progression, metastasis, and drug resistance has also been highlighted^5–8^. Microenvironment can influence miRNA mediated regulation of gene expression in cancer cells either by regulating their expression in tumor cells via modulating signaling networks or by direct miRNA transfer^9^. Identifying the molecular mechanisms and dynamics of microenvironment mediated response to therapy and cancer progression is therefore crucial to improve therapy and achieve prolonged disease free survival in cancer patients.

Acute myeloid leukemia involves abnormal proliferation and accumulation of immature myeloid cells. Currently, World Health Organization (WHO) had classified this disease based on its molecular pathology. Amongst the different AML subtypes, APL (AML-M3) is known to have best response to therapy when arsenic trioxide (ATO) based regimens are used. However, even in APL the relapse rate varies from 10–15% when ATO is used as a single agent. Unlike most cancers it has been noticed that primary resistance to ATO is almost never seen in patients both at initial diagnosis and at relapse^10,11^. This suggests that there could be other mechanisms by which the leukemic cells in APL evade drug toxicity rather than by clonal evolution or drug binding site mutations. Microenvironment-mediated drug resistance or adaptation of leukemic cells is one of the mechanisms where the relapse of the disease can occur through persistence of minimal residual disease. Previous reports, including data from our laboratory suggests that bone-marrow stromal cell mediated drug resistance, predominantly mediated by NF-kB signaling, is significant in APL and AML cell lines and primary cells^12,13^.

In this study, we have uncovered the molecular mechanism of stroma mediated drug resistance via NF-kB signaling in APL. We find that stromal cells activate NF-kB signaling in leukemic cells which is directly responsible for downregulation of *miR-23a-5p*. Downregulation of *miR-23a-5p* in co-cultured leukemic cells results in upregulation of protective autophagy via TLR2, which protects the leukemic cells from chemotherapy induced apoptosis. Using GFP-based miRNA reporter constructs and *miR-23a-5p* mimic, we demonstrate that this miRNA plays a significant role in protection of leukemic cells against chemotherapy toxicity. We also demonstrate that this molecular mechanism of drug resistance identified in APL, is also relevant in some AML cell-lines and patient samples but not in acute lymphoid leukemia.

## Results

### Malignant promyelocytes upon interaction with bone-marrow stromal cells significantly downregulates miR-23a-5p

**Fig. 1:**
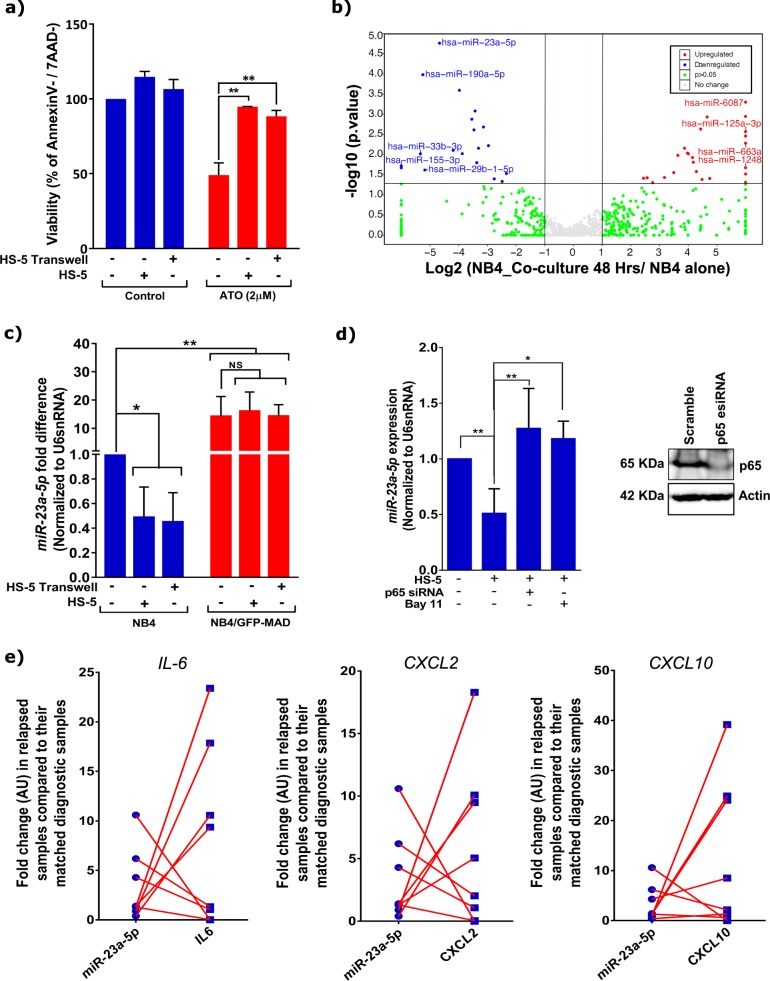
Bone-marrow stromal cells protects leukemic cells from chemotherapy induced apoptosis via NF-kB pathway mediated suppression of *miR-23a-5p* expression. **a** Stromal cells induces a protective effect against arsenic trioxide in malignant promyelocytes (NB4) in both contact dependent and independent systems (*n* = 5) Viability was assessed using Annexin V /AAD kit, post 48 h treatment with ATO, the viability of untreated cells were normalized to 100% and the treated cells viability were compared to normalized untreated cells. **b** Volcano Plot represents fold change in miRNA content in NB4 cells upon co-culture with stromal cells. Arrow represents the miRNA of interest which as changed to significant levels (*p* < 0.05). We plotted volcano plot using R ggplot2 package. **c** *miR-23a-5p* in leukemic cells (NB4) is downregulated upon co-culture (direct and transwell) with stromal cells and NB4/GFP-MAD cells showing high expression of *miR-23a-5p* compared to NB4 cells. Downregulation of *miR-23a-5p* was not observed in NB4/GFP-MAD cells even after co-culture with stromal cells NB4/GFP-MAD cells showing high expression of *miR-23a-5p* compared to NB4 cells (*n* = 3). **d** *miR-23a-5p* in leukemic cells is downregulated on co-culture with stromal cells and this effect is reversed on inhibiting the NF-kB pathway as demonstrated here by either knock down of p65 or by use of small molecule inhibitors of the NF-kB pathway (bay-11; 10 µM) (*n* = 3). **e** NF-kB target genes (*IL-6, CXCL2, CXCL10*) levels in the relapsed (compared to their matched diagnostic samples) inversely correlated with *miR-23a-5p* levels for the same samples at relapse. Statistical significance was calculated using Student’s *t-*test (two tailed *t-*test) and the values are denoted as mean ± SD. The *P-*values are denoted as *P = 0.02, **P = 0.005, ****P < 0.0001, NS Not significant

Leukemic cell-lines, as well as the primary blasts from APL patients demonstrate survival advantage against ATO when co-cultured with either primary stromal cells or stromal cell-lines^14^. This stroma-mediated protective effect against ATO is both contact dependent and independent (Fig. 1a and supplementary Fig. 1). Since miRNAs are known to be one of the major regulators of therapy-resistance in different cancers, we focused on deciphering if cellular miRNAs are differentially expressed in leukemic cells upon stromal co-culture to mediate this protective effect. Towards this, we analyzed the expression of miRNAs in leukemic cells with and without stromal co-culture. Several miRNAs were differentially expressed in leukemic cells after stromal co-culture (supplementary Table 1). miRNAs which have been validated for their role in inducing apoptosis^15–19^ were downregulated; while the miRNAs known to be involved in anti-apoptosis mechanism^20–22^ were upregulated in the co-cultured leukemic cells (Fig. 1b). Among these differentially regulated miRNAs, we found that *miR-23a-5p* was the most significantly downregulated and stood out even after employing stringent analysis parameters using Deseq (supplementary Fig. 2 and supplementary Table 1) and we could validated its downregulation by Q-PCR analysis (Fig. 1c). Moreover, *miR-23a-5p* can act as both oncogene and tumor suppressor^23,24^, hence we selected *miR-23a-5p* to further evaluate its role in stromal cells-induced ATO-resistance.

### Downregulation of miR-23a-5p in leukemic cells correlates with upregulation of NF-kB pathway

NF-kB pathway is known to play an important role in stromal cell mediated protective effect^12^. In our current experiments too we were able to confirm NF-kB upregulation in leukemic cells upon stromal co-culture (supplementary Figs. 3 and 4). To evaluate if *miR-23a-5p* expression could be regulated by NF-kB signaling or vice-a-versa, we took a variant of NB4 cell-line (NB4/GFP-MAD cells) where the NF-kB pathway was repressed by overexpressing a mutant IkB super-repressor (supplementary Fig. 5). We found that NB4/GFP-MAD cells showed no significant alteration in the levels of *miR-23a-5p* upon stromal co-culture (Fig. 1c). Expression of *miR-23a-5p* was also significantly higher in NB4/GFP-MAD compared to NB4 (Fig. 1c). This inverse correlation between NF-kB signaling and *miR-23a-5p* suggests that NF-kB pathway regulates *miR-23a-5p* expression. To further resolve the relationship between NF-kB and *miR-23a-5p*, we also inhibited NF-kB signaling using Bay11 or p65 esiRNA in NB4 cells, this again lead to upregulation of *miR-23a-5p* levels in leukemic cells (Fig. 1d). Our results thus suggests that the activation of NF-kB pathway via stromal interactions (contact dependent or independent) negatively regulates the expression of *miR-23a-5p* in leukemic cells. This inverse relationship between *miR-23a-5p* and NF-kB signaling was also evident in APL patient’s samples, as assessed by NF-kB target gene expression (*CXCL2, CXCL10, IL6*) and *miR-23a-5p* expression (Fig. 1e).

### Stroma-mediated downregulation of miR-23a-5p can drive drug-resistance and relapse in APL

**Fig. 2:**
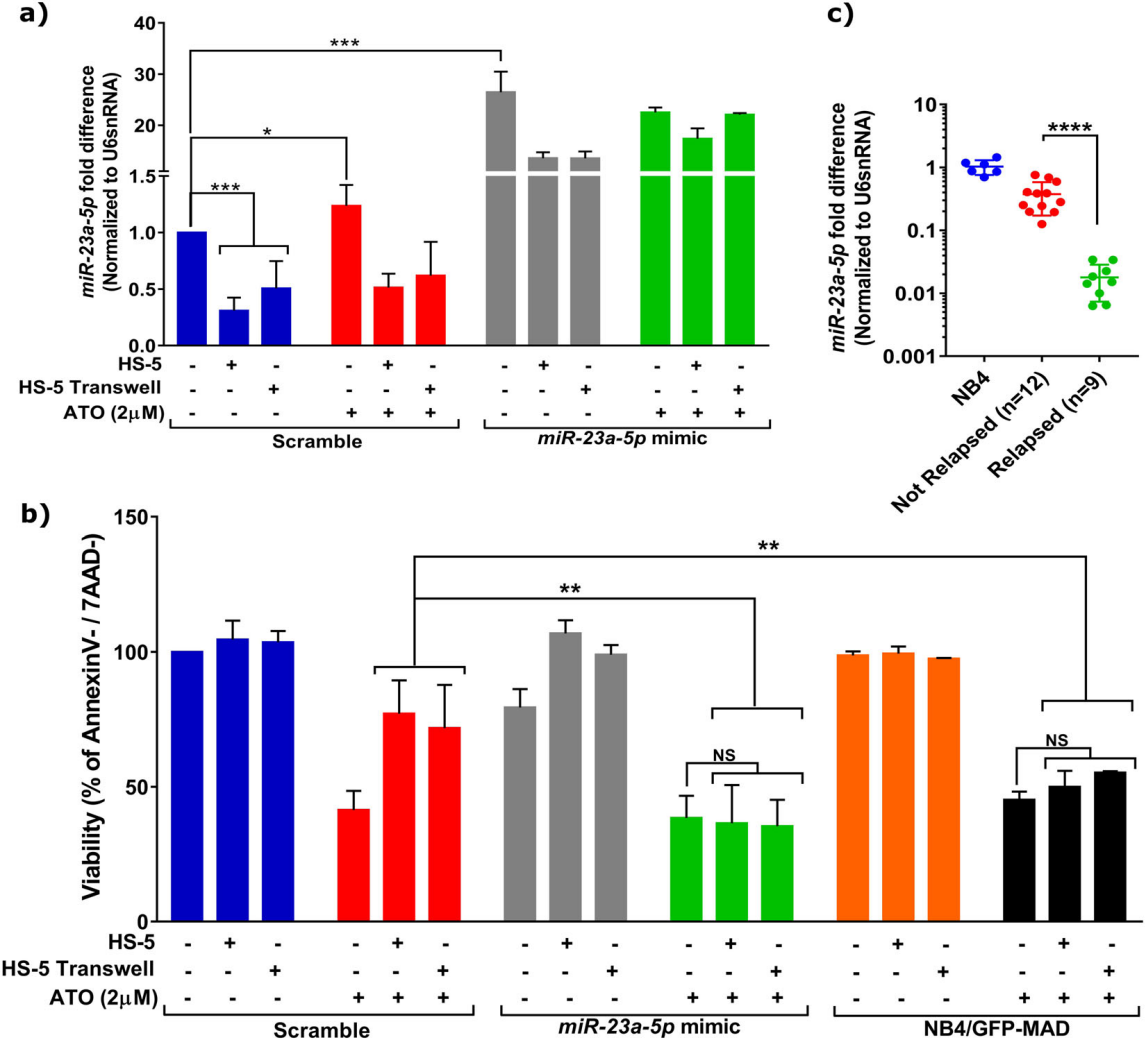
Stromal cells mediated downregulation of *miR-23a-5p* is responsible for drug-resistance **a** Effect of ATO on *miR-23a-5p* expression in leukemic cells upon co-culture, as well as during mimic overexpression conditions (*n* = 3). **b** Re-introduction of *miR-23a-5p* mimic into NB4 cells restores sensitivity against ATO in the presence of stroma (*n* = 3) in both transwell and direct co-culture conditions, viability was measured using Annexin V/7AAD kit. NB4/GFP-MAD cells having high expression of miR-23a-5p did not show a protective effect against ATO in the presence of stroma. **c** Correlation of expression of *miR-23a-5p* in APL patients to relapse. Expression *miR-23a-5p* (normalized with U6snRNA-housekeeping) and compared to NB4 cell-line, suggesting higher expression of *miR-23a-5p* in patients who had not relapsed compared to patients who had relapsed. Statistical significance was calculated using Student’s *t-*test (two tailed *t-*test) and the values are denoted as mean ± SD. The *P*-values are denoted as **P = 0.005, ***P < 0.001, ****P < 0.0001, NS not significant

Next, we analyzed the expression of miR-23a-5p in NB4 cells upon treatment with ATO and we noted that ATO significantly increased the expression of miR-23a-5p levels (Fig. 2a). Moreover, we noted a modest increase in the expression of this miRNA when the cells were in co-culture and treated with ATO compared to co-culture alone (Fig. 2a). Further, to investigate if downregulation of *miR-23a-5p* in leukemic cells during stromal co-culture was responsible for drug-resistance, we overexpressed *miR-23a-5*p in NB4 cells using mimics. Overexpression of *miR-23a-5p* mimics was confirmed by Q-PCR (Fig. 2a), as well as using GFP-*miR-23a-5p*-reporter assay (supplementary Fig. 6). Overexpression of *miR-23a-5p* mimic, restored sensitivity to ATO (Fig. 2b and supplementary Fig. 7) and daunorubicin (DNR) (supplementary Fig. 8) in NB4 cells even in the presence of stromal co-culture. Also, NB4/GFP-MAD cells which show higher cellular *miR-23a-5p* levels were sensitive to ATO (Fig. 2b) and DNR even in presence of stroma cells (supplementary Fig. 8). We also analyzed the expression of *miR-23a-5p* in primary APL samples collected at the time of diagnosis and correlated with relapse. We observed a significantly lower expression of *miR-23a-5p* in ‘at diagnosis’ sample of patients who had relapsed subsequently (Fig. 2c) compared to patients who did not relapse upon ATO treatment. Together our results demonstrate that the restoration of cellular *miR-23a-5p* levels could overcome stroma-mediated protection against chemotherapeutics drugs in leukemic cells and downregulation of this miRNA can be correlated to relapse in APL.

### miR-23a-5p targets TLR2 and modulates autophagic flux in leukemic cells

**Fig. 3:**
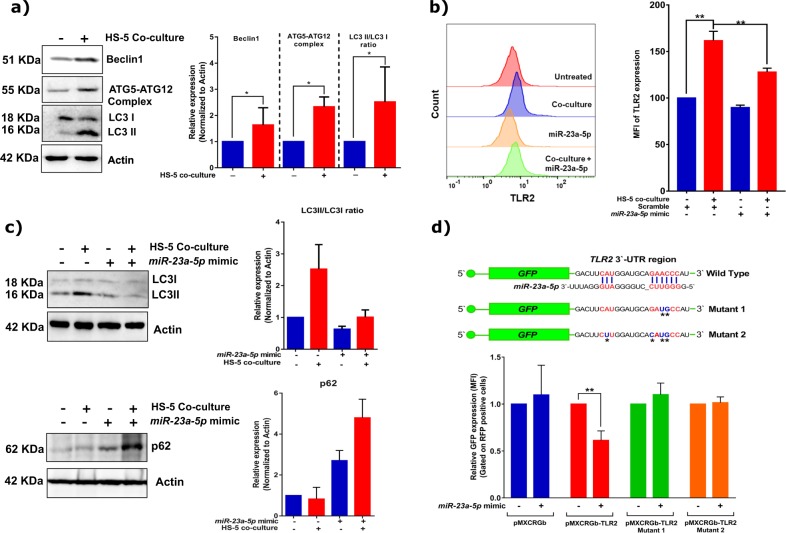
Stromal cells mediated downregulation of *miR-23a-5p* induces drug-resistance by targeting TLR2 expression: **a** Autophagy is upregulated in malignant promyelocytes upon co-culture with stromal cells. Western blots showing the protein intensities were quantitated by densitometry and normalized to actin (*n* = 3). **b** Expression of TLR2 (protein) in leukemic cells upon co-culture and in the presence of *miR-23a-5p* mimic (*n* = 3) by a flow cytometry based assay. **c** Autophagic flux as measured by decrease levels in p62 levels and increase levels of LC3II is upregulated in malignant promyelocytes upon co-culture with stromal cells and *miR-23a-5p* targets autophagic flux as shown by accumulation of p62 in malignant promyelocytes upon co-culture (*n* = 3). Western blots showing the protein intensities were quantitated by densitometry and normalized to actin (*n* = 3). **d** Top panel-cartoon showing the 3′UTR region of *TLR2* mRNA where *miR-23a-5p* can bind as well the mutant showing the modified bases. Quantitation of GFP fluorescence (Mean Flourescent Intensity-MFI) when *miR-23a-5p* is re-introduced in NB4 cells which were made to transiently express GFP protein where the 3′UTR of GFP is replaced by TLR2 3′UTR (pMXCRGb-TLR2). This reduction in GFP intensity was not observed in control system having vector alone (pMXCRGb) or in the mutants generated (*n* = 3). Statistical significance was calculated using Student’s *t-*test (two tailed *t-*test) and the values are denoted as mean ± SD. The *P*-values are denoted as *P = 0.02, **P = 0.005

To get functional insights about the role of *miR-23a-5p* in mediating drug sensitivity, it becomes imperative to identify its targets. Using TargetScan-software the genes involved in autophagy and toll-like receptors (TLR) were identified as top five targets that could be potentially regulated by *miR-23a-5p* (supplementary Table 2). We observed that in co-cultured leukemic cells where *miR-23a-5p* is downregulated, there was increase in autophagy proteins, as well as TLR2 (Fig. 3a, b). To identify if TLR2, as well as autophagy proteins were direct targets of *miR-23a-5p*, we evaluated levels of TLR2 and autophagy genes in miRNA add-back conditions. We noted decreased expression of TLR2 protein, as well as TLR2 transcript (Fig. 3b and supplementary Fig. 9) and decreased autophagic flux (as measured through accumulation of cellular levels of p62/SQSTM1 protein and LC3-II conversion; Fig. 3c). However, the mRNA levels of autophagy genes remained unperturbed (supplementary Fig. 10). NB4/GFP-MAD cells, where *miR-23a-5p* levels are significantly higher, also demonstrated decreased expression of TLR2 (supplementary Fig. 11). To validate that TLR2 is a direct target of *miR-23a-5p* in leukemic cells, we generated a GFP-reporter by cloning 3′UTR of *TLR2* region (identified by TargetScan software as *miR-23a-5p* target) at 3′end of GFP (Fig. 3d, top panel). *miR-23a-5p* mimic when overexpressed in leukemic cells could lead to decrease in the intensity of GFP-TLR2-reporter expressing cells unlike the control or the TLR2–3′UTR mutants (Fig. 3d). Overall our data suggests that TLR2 is the direct target of miR-23a-5p.

### TLR2 expression regulates autophagy levels and induces ATO-resistance in APL

**Fig. 4:**
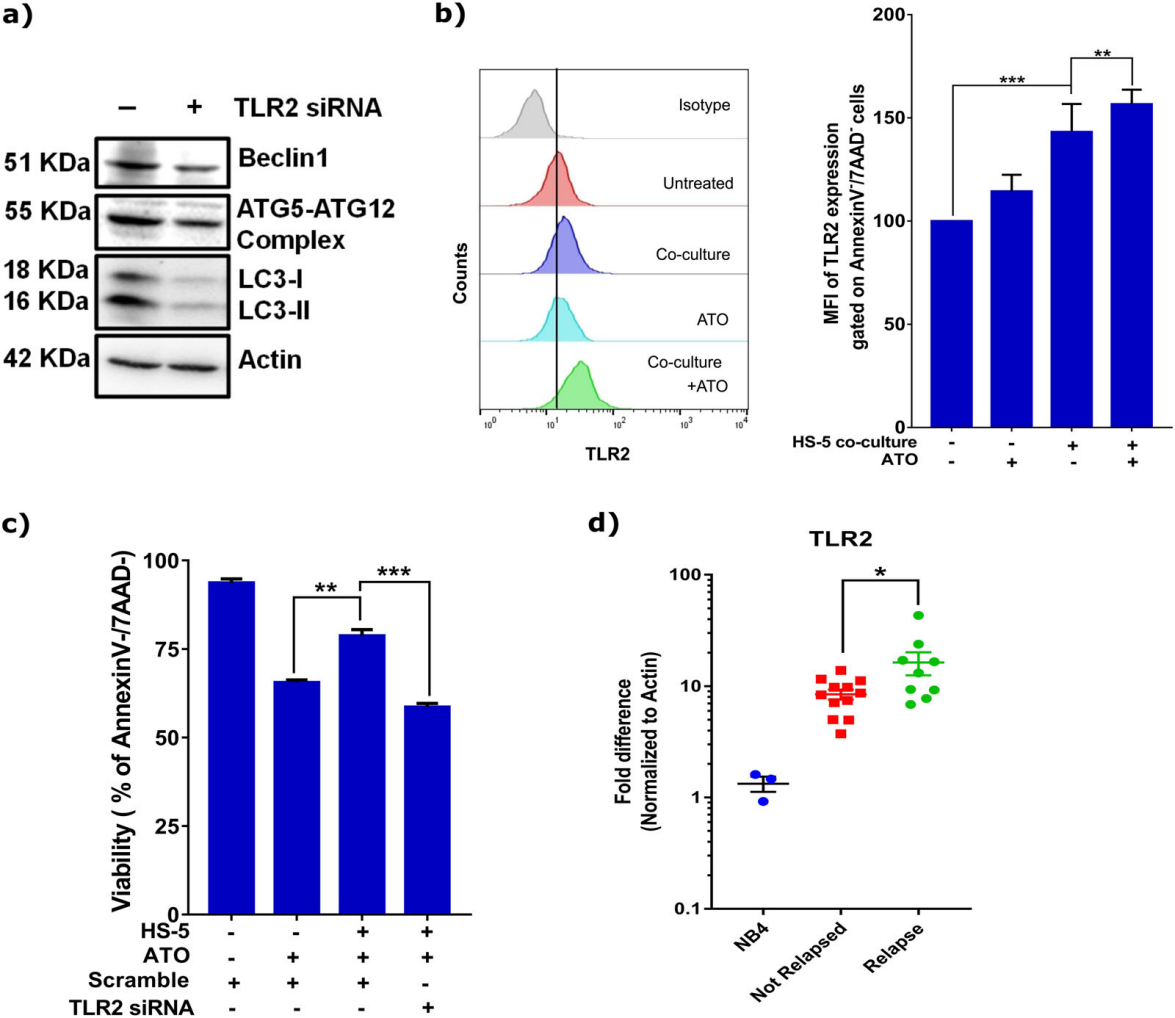
m*iR-23a-5p* targets autophagic flux and autophagy inhibitors can overcome stroma mediated protection to ATO. **a** Autophagy is regulated by TLR2 – knock down of TLR2 gene in NB4 resulted in downregulation of genes (*LC3, Beclin1, ATG12*) involved in autophagy pathway (*n* = 3). **b** Expression (MFI) of TLR2 on NB4 cells which are surviving (Annexin V-/7AAD-) post ATO exposure (2 µM) in the presence of stromal cells shows increased expression of TLR2 (n = 3) compared to controls. **c** Transient knock down of TLR2 sensitized the NB4 cells to ATO even in the presence of stromal cells; viability assay was performed using AnnexinV and 7AAD (*n* = 3). **d** Correlation of expression of TLR2 in APL patients to relapse. Expression of TLR2 (normalized with actin-housekeeping) were compared to NB4 cell-line, suggesting higher expression of TLR2 in patients who had relapsed compared to patients who had not relapsed). Statistical significance was calculated using Student’s *t-*test (two tailed *t-*test) and the values are denoted as mean ± SD. The *P*-values are denoted as *P = 0.02, **P = 0.005, ***P < 0.001

TLR2 is known to regulate autophagy^25,26^. We hence hypothesized that decrease in autophagic flux might be influenced by TLR2. To evaluate this, we transiently knocked-down *TLR2* in NB4 cells. Knock-down of *TLR2* resulted in decreased expression of autophagy proteins (LC3, ATG5–12, and Beclin1) (Fig. 4a), even in presence of stromal-cells (supplementary Fig. 12). Increased expression of TLR2 was also observed in co-cultured NB4 cells post ATO-exposure (Fig. 4b) suggesting that TLR2 could play an important role in drug-resistance. Further, the *TLR2-*knowdown NB4 cells were sensitized to ATO in the presence of stromal cells (Fig. 4c and supplementary Fig. 13). Further, a modestly upregulated expression of *TLR2* in APL patients at diagnosis was noted in those that subsequently relapsed (Fig. 4d). Altogether, we observed that stromal cells induced downregulation of *miR-23a-5p* in leukemic cells can increase TLR2 expression which in turn regulates autophagy pathway to induce drug-resistance.

### miR-23a-5p also targets TLR2 and autophagy in other subtypes of acute myeloid leukemia

**Fig. 5:**
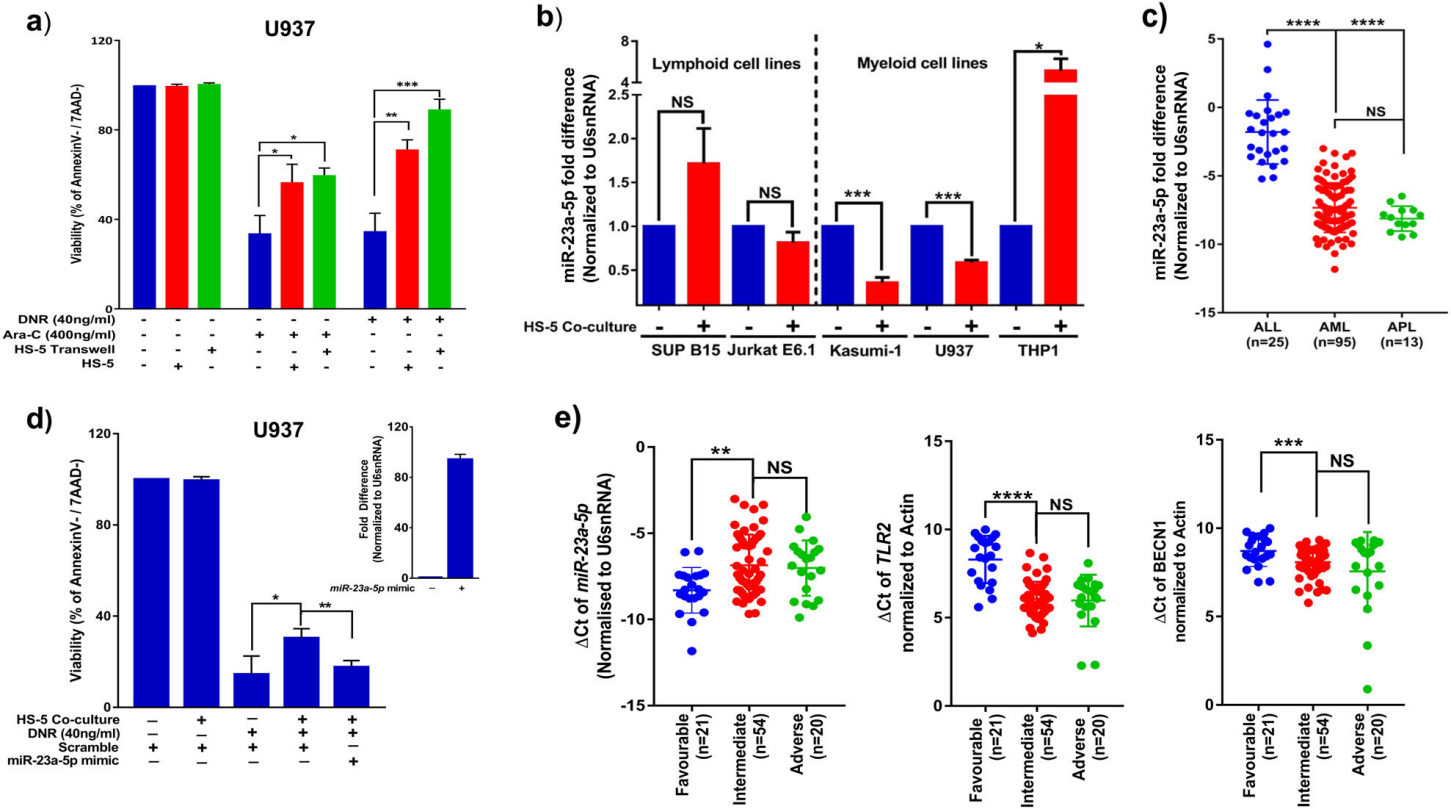
m*iR-23a-5p* also targets TLR2 and autophagy flux in other subtypes of acute myeloid leukemia: **a** Stromal cells induces a protective effect against arsenic trioxide in AML cell-line (U937) in both contact dependent and independent systems (*n* = 3). Viability was assessed using Annexin V /AAD kit, post 48 h treatment with daunorubicin (DNR-40ng/ml) and cytarabine (Ara-C-400 ng/ml). The viability of untreated cells were normalized to 100% and the treated cells viability were compared to normalized untreated cells. **b** Expression of *miR-23a-5p* (2-ΔΔCt-fold difference) in various lymphoid and myeloid leukemic cells co-cultured with stromal cells (HS-5) (*n* = 3). **c** Expression (ΔCt) of *miR-23a-5p* in primary ALL (*n* = 25), AML (*n* = 27) and APL (*n* = 15) samples (ΔCt is inversely correlated to the expression). **d** Re-introduction of *miR-23a-5p* mimic into U937 cells restores sensitivity against DNR in the presence of stroma (*n* = 3), viability was measured using Annexin V/7AAD kit. Insert showing the efficacy of electroporation in U937 cells (*n* = 3). **e** Higher expression of *miR-23a-5p* and lower expression of *TLR2* and *BECN1* correlates with favorable risk prognosis (*n* = 21) in AML (including AML-M3; *n* = 13). Lower expression of *miR-23a-5p* and higher expression of *TLR2* and *BECN1* also correlated with intermediate (*n* = 54) and adverse risk (*n* = 20) AML patients. (ΔCt values are inversely proportional to the expression levels of genes). Statistical significance was calculated using Student’s *t-*test (two tailed *t-*test) and the values are denoted as mean ± SD. The *P*-values are denoted as ***P = 0.0001, **P = 0.005, *P = 0.02, NS not significant

Stromal cell mediated drug-resistance has also been implicated in other subtypes of AML. We hence investigated if HS-5 cells could also protect different AML cell-lines against their respective chemotherapeutic agents. We observed significant protection in co-cultured AML cell-lines (U937, THP1, Kasumi-1) and primary AML cells against DNR or Cytarabine (Ara-C) (Fig. 5a and supplementary Fig. 14). Activation of NF-kB pathway was also observed in AML cells upon co-culture (supplementary Fig. 15). Further, compared to NB4 cells; NB4/GFP-MAD and Kasumi-1 cell-lines had higher expression of *miR-23a-5p* while myeloid leukemic cells such as U937, HEL, and THP1 cell-lines had lower expression of miR-23a-5p (supplementary Fig. 16). Interestingly, we noted that this miRNA was downregulated even in NB4 EV-AsR1(supplementary Fig. 16; an in-house derived ATO resistant cell-line). Further we looked for the expression of *miR-23a-5p* in different AML cells upon co-culture. Downregulation of this miRNA upon stromal co-culture was seen with U937 and Kasumi-1 (AML cell-lines) but not in THP-1 (AML cell-line) or the Sup-B15 and Jurkat-E6.1 cell-lines (ALL cell-line; Fig. 5b). We identified that there was also significant difference in levels of *miR-23a-5p* between ALL, AML, and APL patients (Fig. 5c).

Similar to APL, we also noted an increase expression of this miRNA in U937 cells when exposed to daunorubicin (supplementary Fig. 17). Further, when the *miR-23a-5p* mimics were re-introduced in AML cells, there was a reversal of resistance in U937 cell-line but not in THP1 cell-line against DNR (Fig. 5d and supplementary Fig. 18). Also, upregulation of *TLR2* upon stromal co-culture was observed only with U937 but not with THP-1 cells (supplementary Fig. 19). The difference in the regulation of TLR2-expression by *miR-23a-5p* overexpression suggests that while stroma-mediated protection is observed in most leukemic cells; the molecular mechanism of this protection is variable and needs to be identified in a context specific manner.

Further, to evaluate if the *miR-23a-5p-*TLR2-autophagy circuit can be used to predict the outcome in patients we correlated the expression of *miR-23a-5p* with prognosis (favorable risk, intermediate risk and adverse risk) in AML patients. Our preliminary results showed that, as observed in APL patient samples (Fig. 2b), high expression of *miR-23a-5p* correlated with favorable risk AML patients; while there were no differences in *miR-23a-5p* expression in intermediate and adverse risk patients (Fig. 5e). Once again, we also noticed a reduction in expression of *TLR2* and *BECN1* (autophagy gene) in favorable risk AML samples when compared to intermediate or adverse risk patient’s samples (Fig. 5e).

### Inhibition of autophagy can overcome stroma-mediated drug-resistance in vitro and modestly improve survival in an APL transplantable mouse model

**Fig. 6:**
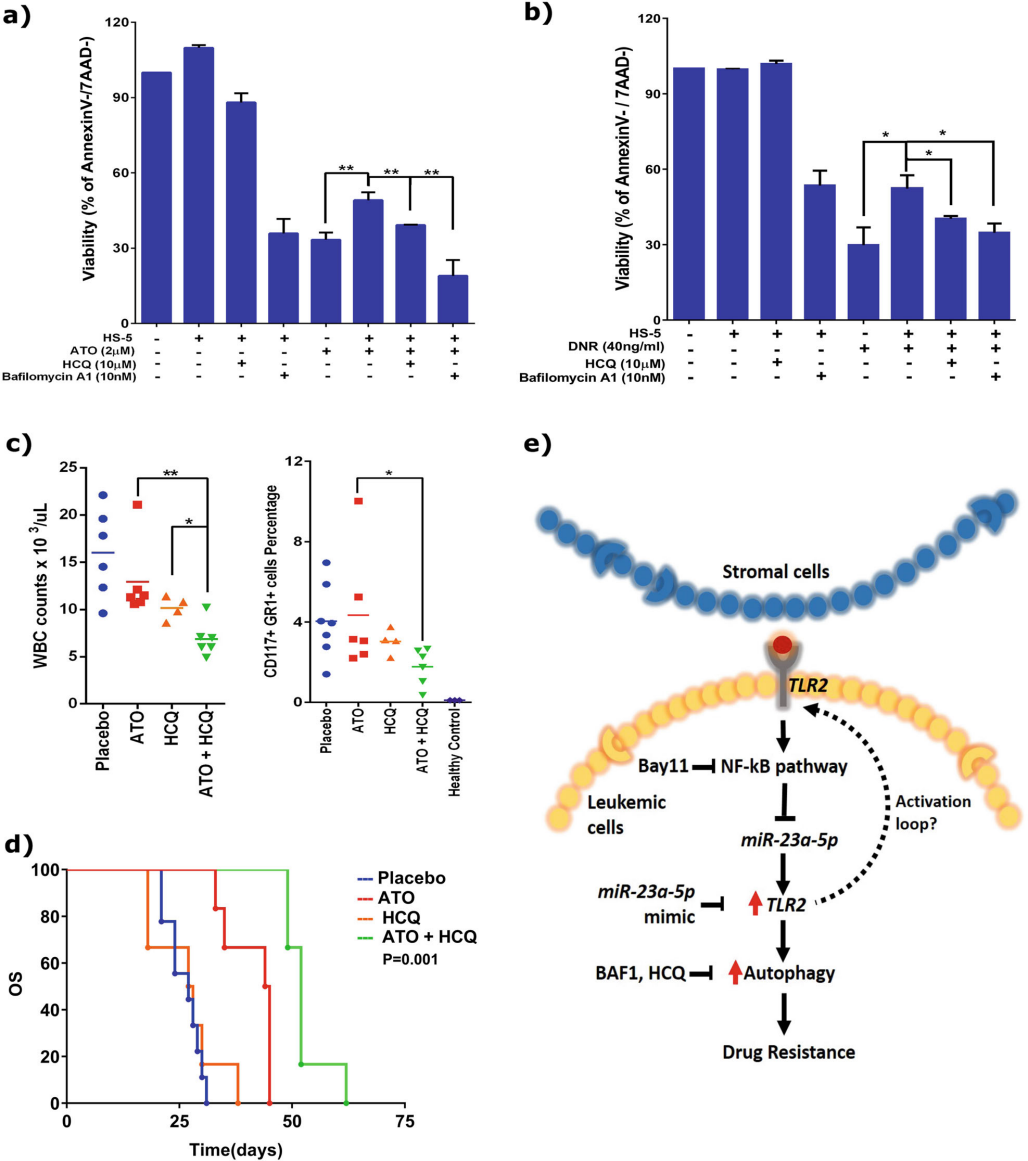
Inhibition of autophagy restores sensitivity to chemotherapeutics drugs in acute myeloid leukemia **a, b** Inhibition of autophagy using autophagy inhibitors (bafilomycin A1–10 nM and hydroxychloroquine–10 µM) restores the sensitivity of ATO (2 µM) in NB4 cells (**a**) and DNR in U937 (**b**) in the presence of stroma (*n* = 3), assays were done at the end of 48 h using apoptosis assay (AnnexinV /7AAD kit). **c** ATO along with HCQ reduces tumor burden in APL mice on day 20 as evidenced through decreased WBC counts and decreased leukemic cells percentage in peripheral blood analyzed by flow cytometry. **d** ATO and HCQ prolongs the survival of APL mice as illustrated in the Kaplan–Meier plot. **e** Image representing the stromal cell mediated downregulation of *miR-23a-5p* and its effect on drug-resistance in leukemic cells. Statistical significance was calculated using Student’s *t-*test (two tailed *t-*test) and the values are denoted as mean ± SD. The *P*-values are denoted as **P = 0.005, *P = 0.02, NS Not significant

Since we identified NF-kB-*miR-23a-5p*-TLR2-autophagy circuit can drive drug-resistance, we evaluated if inhibition of autophagy (activated due to low *miR-23a-5p* levels) is sufficient to overcome stroma-mediated protective effect. For this, we treated co-cultured cells with known autophagy inhibitors-Bafilomycin A1 and Hydroxychloroquine (HCQ) along with ATO. The ability of the autophagy inhibitor (at the concentrations used) to inhibit autophagy was verified (supplementary Fig. 20). As observed with *miR-23a-5p* mimic overexpression condition, autophagy inhibitors were also able to restore the sensitivity of ATO in APL, as well as to DNR in AML cells upon co-culture (Fig. 6a, b and supplementary Figs. 21 and 22). Taken together, our results demonstrates that the stroma mediated upregulation of protective-autophagy in leukemic cells, via downregulation of cellular *miR-23a-5p* levels, results in protection against conventional therapy in vitro. Finally, we evaluated the efficacy of HCQ along with ATO to improve the overall survival in an APL mouse model. Based on our previous experience^12^, we used reduced dosage of ATO (from 10 mg/kg to 5 mg/kg) so that a synergy between ATO and HCQ, if any, could be observed. We find that when HCQ was combined with ATO, there was significant reduction of tumor burden in the APL mice (Fig. 6c, supplementary Fig. 23) in the peripheral blood of APL mice on day 20 compared to controls. Though, this combination showed a significant decrease in the leukemia burden, there was modest improvement in the survival of APL mice (median 52 days) when compared to placebo (median 27 days), ATO (median 45 days) or HCQ (median 27.5 days) arms (Fig. 6d). Our results here, using cell based assays, as well as APL mouse model, suggests that inhibiting protective autophagy can overcome the resistance induced by stroma in APL. Our data also suggests that, NF-kB-*miR-23a-5p-*TLR2-Autophagy axis is an important target to overcome drug-resistance in APL and subtypes of AML (Fig. 6e).

## Discussion

Accumulating evidence suggests that bone marrow stromal micro-environment act as sanctuary site for leukemic cells and affords protection from conventional chemotherapy agents. We had previously reported in APL that there was significant microenvironment-mediated ATO-resistance by upregulation of the NF-kB pathway^12^. In this manuscript we have addressed the molecular mechanism of NF-kB pathway mediated drug-resistance in AMLs. Here, using in vitro co-culture assays we try to mimic the intercellular cross talk conditions. To identify the secreted factor(s) involved in activation of NF-kB signaling, we performed cytokine array using conditioned medium from stromal cells line. Our results suggests that several cytokines, up to 43, were upregulated upon stromal co-cultures (data not shown), several of which could potentially activate NF-kB signaling. Apart from cytokines, exosomes derived from stromal cells are also known for their protective effect on the cancer cells via juxtacrine signaling or due to delivery of exosomal miRNAs or other regulatory factors ferried in the lumen of the exosomes^27,28^. Recently, even Wnt5 has also been identified as a ligand involved in activate NF-kB signaling^29^. Wnt proteins are also known to be ferried on exosomes^30^. Thus NF-kB signaling can be activated by several possible stroma-derived paracrine factors. Secreted cytokines and exosomes can act in additive or redundant manner to activate NF-kB signaling. A more detailed study needs to be initiated to understand if NF-kB activation or ATO resistance can be imparted by stromal cells in exosome dependent or independent manner and to nail the dominant player (if any) in activation of NF-kB signaling in leukemic cells upon stromal co-culture.

It has been reported that apart from upregulating cell proliferation and anti-apoptotic genes, NF-kB signaling is also known to exhibit their pro-tumorigenic activity by regulating expression of specific miRNAs^31,32^. Here, using NGS based approach and stringent analysis parameters, we identify that *miR-23*a-5p is significantly downregulated among other miRNAs in co-cultured leukemic cells. Our data using NF-kB inhibitors, NF-kB signaling incompetent leukemic cells, as well as relapsed APL patient samples, strongly suggest that downregulation of *miR-23a-5p* is a result of upregulated NF-kB signaling upon stromal co-culture. This inverse relationship between *miR-23a-5p* and NF-kB identified in leukemic cells by our study is in agreement with a study done in Jurkat cells, where NF-kB has been shown to inversely regulate miR-23a-5p expression^33^; unlike HEK, K562 and HEL cells were NF-kB signaling upregulated expression of *miR23a-27a-24* cluster^34^. The observed difference in regulation of *miR-23a-5p* levels suggests role of other tissue specific regulators.

Further, *miR-23a-5p* overexpression experiments suggests that downregulation of this miRNA plays a significant role in protection of leukemic cells against chemotherapy toxicity. To the best of our knowledge this is the first time that *miR-23a-5p* has been implicated in leukemia drug-resistance in a NF-kB dependent manner. In order to understand the molecular mechanism of *miR-23a-5p* downregulation mediated drug-resistance in AML cells, we predicted the targets of *miR-23a-5p*. As reported for Mycobacterium infected macrophages^35^, we were also able to validate *TLR2* as its direct target using GFP-reporter assays and *miR-23a-5p* mimics. Further knock-down of *TLR2* resulted in reversal of drug-resistance in leukemic cells even in the presence of stroma. Consistent with our findings, it has also been reported that increased expression of TLR2 in AML is associated with poor clinical outcomes^36^.

Overexpressing *miR-23a-5p* in leukemic cells results in downregulation of cellular autophagic flux, estimated by accumulation of p62 [a well-established autophagy target^37^], as well as reduced the expression of autophagy proteins. Here, we conclude that this *miR-23a-5p-*mediated effect on autophagy pathway is via TLR2 since overexpression of *miR-23a-5p* mimics resulted in downregulation of *TLR2* transcripts but did not influence autophagy transcripts. Therefore, TLR2 may activate cellular autophagy probably via post translational modifications of autophagy genes^38^. Upregulation of autophagy and its association with drug-resistance identified here, is consistent with reported clinical studies in AML that have demonstrated an increased risk of relapse and decreased overall survival associated with an increased expression of autophagy related genes^39,40^.

Further, we observed that both, HCQ or bafilomycin A1 when used in combination with DNR or ATO can reverse the stroma-mediated drug protection and can improve survival in APL mouse model. This suggests that autophagy inhibitors when used as adjuvants can overcome stroma-mediated protection to leukemic cells in vitro, as well as in vivo conditions (APL mouse model) for better treatment outcome. It must be noted that we had demonstrated previously that bortezomib when used in combination with ATO can improve therapy outcome by inducing cytotoxic-autophagy^12^. Whereas this study suggests that the stromal co-culture induces cytoprotective-autophagy in leukemic cells. It is well noted that autophagy is a ‘double edged sword’ and the threshold where the protective-autophagy can be converted into cytotoxic-autophagy is not known. However, nuclear accumulation of B-catenin is a known marker of cytotoxic-autophagy^41^; we found that the autophagy induced during drug treatment [based on our previous study^12^], indeed leads to nuclear accumulation of B-catenin unlike stromal co-cultures (supplementary Fig. 24). Based on these observations we conclude that, in contrast to bortezomib, stromal co-cultures activate protective-autophagy in these cells.

Further, in order to evaluate impact of our findings in other leukemia’s, we attempted to validate if the *miR-23a-5p-*mediated protective influence is valid for myeloid (NB4, U937, Kasumi-1, THP-1) and lymphoid cells (Jurkat E6.1 and SUP B15). We were able to demonstrate a significant downregulation *of miR-23*a-5p in myeloid malignant cells (except THP1) but not in lymphoid cells upon co-culture. Data from primary cells suggested that at baseline APL cells had the highest expression *of miR-23*a-5p followed by AML (with variable expression levels) and ALL samples, respectively. Comparing the conventionally defined risk groups in AML, the favorable risk group patients had highest expression *of miR-23*a-5p compared to intermediate and adverse risk groups analyzed. The high expression of *miR-23a-5p* could be one of the reason why APL cells are most sensitive to ATO and anthracycline and being most curable leukemia, while other acute leukemia are not. Further, correlating relapse with *miR-23a-5p* expression, we identified that those who relapsed had a lower expression of *miR-23a-5p* in their malignant cells at diagnosis compared to those who did not relapse. We suspect that there will be a significant heterogeneity in the cellular levels of miR-23a-5p in AML patient samples and active regulation of miR-23a-5p-TLR2-Autophagy circuit which might be restricted to rather small subset of cells which are close to the bone-marrow stromal cells which would in turn contribute to disease relapse. While upregulation of autophagy in AML cells upon co-culture has been identified before^42,43^, our work resolves the underlying molecular events, as well as their dynamic nature. It must be noted that, the illustrated stroma-mediated mechanism of protection against chemotherapeutic agents was predominantly seen in myeloid leukemia it cannot be generalized to all myeloid leukemia’s, as noted with exception seen with THP1 cell-line.

This study thus demonstrates that myeloid leukemia cells can adapt themselves to the drug-induced stress by interacting with stromal cells in bone-marrow niches. Identifying and targeting such molecular cross talks could potentially prove to be an effective strategy in treating high risk or relapsed patients.

## Materials and methods

### Cell-lines and patient samples

The human leukemic cell-line such as U937, Kasumi-1, THP-1, Jurkat E6.1, SUP B15 and stromal cell line HS-5 were obtained from ATCC, USA, NB4 was a kind gift from Dr. Harry Iland, RPAH, Sydney, Australia (with permission from Dr. Michel Lanotte), NB4/GFP and NB4/GFP-MAD cells (Kind gift from Dr. Christine Chomienne, Hôpital St. Louis, Paris, France with permission form Dr. F. Besancon) were used in this study. Mycoplasma detection was done once in every 6 months and all the cell-lines used were free from Mycoplasma. The study was approved by the institute review board (IRB Min. No. 7826 dated 18.04.2012). AML, ALL, APL patient samples at diagnosis and relapse were collected, prior to treatment, after getting written and informed consent.

### Reagents and antibodies

ATO, a kind gift from Intas Pharmaceuticals Ltd, Ahmedabad, India, was used in the study. Daunorubicin, Cytarabine, Bafilomycin A1, Hydroxychloroquine, Bay11-7086 was procured from Sigma, St. Louis, USA. Antibodies used included those against Actin, ATG12, Beclin1, p62, p65 (Santa Cruz, CA, USA), LC3 (Cell Signaling Technology Inc, Massachusetts, USA), TLR2 (BD Pharmingen, New Jersey, USA) anti-mouse and anti-rabbit secondary antibodies conjugated with horseradish peroxidase (Cell Signaling Technology Inc., Massachusetts, USA) and with alexaflour 488 and 594 (Invitrogen, California, USA) were used for western blotting and immunofluorescence.

### Assays for apoptosis

Leukemic cell-lines were added (1 × 10^5^ cells/well) on a layer of primary stromal cells or HS-5 stromal cell-line in 24 well plates or seeded in transwells plates. The co-cultured cells were incubated overnight and then exposed to various chemotherapeutic agents along with appropriate controls. After 48 h incubation at 37 °C CO_2_ incubator, the leukemic cells were carefully pipetted out and their viability was measured using Annexin V/7AAD apoptosis assay kit (BD Pharmingen, New Jersey, USA) as per manufacturer’s protocol. CD105 staining was used to exclude stromal cells if present during acquisition and analysis. The flow data were analyzed using Cell Quest pro software (BD Biosciences, New Jersey, USA).

### Small RNA library preparation and sequencing

Briefly, the NB4 cells were co-cultured with and without HS-5 cells for 24 and 48 h prior to this experiment. Three independent experiments were carried out to serve as biological triplicates. The NB4 cells (control) and the NB4 co-cultured (treated) were compared to generate statistically significant differentially expressed small RNAs. Small RNA libraries were prepared using the Illumina TruSeq small RNA kit as described by the manufacturer (Illumina). 1 microgram of total RNA were used from each sample for the library preparation. 5' and 3' Small RNA adaptors were ligated to the RNA and the ligated products were reverse transcribed using superscript II reverse transcriptase (Invitrogen, California, USA). The RT products were then amplified by PCR and resolved on an 8% polyacrylamide gel. Bands corresponding to 140–160 nucleotides nt were gel eluted. The size and integrity of each library was verified using the Bioanalyser. The libraries were sequenced on an Illumina Hiseq 1000 (Centre for cellular and Molecular Platforms (CCAMP), Bengaluru, India).

### miRNA analysis

From the sequencing reads, we trimmed TruSeq small RNA adapters using customized perl script and cutadapt^44^ program. We then mapped these reads to rRNA database and unaligned reads were taken for further analysis. We then segregated reads that are 18–24 nucleotides and mapped to GRCh38 Genome and miRNA databases^45^ using bowtie v1.0.0^46^ Customized perl script was used to obtain count data for all the miRNAs. The count data was normalized using DESeq^47^ and the normalized data was used for further analysis. miRNAs which have adjusted *p* value < 0.05 were considered for further analysis. We plotted volcano plot using R ggplot2 package. For figure esthetic purpose, miRnas with adj.pvalue 1 are replaced with their *p*-values (if *p* value > 0.05). Targets for differentially expressed miRNAs were predicted using TargetScan v7.1^48^ miRNA targets were overlapped with microarray data to identify targets that are upregulated/not changing. We did pathway and gene Ontology GO analysis of these genes using Gene Set Enrichment Analysis (GSEA)^49^.

### miRNA RT-PCR

Quantitative PCR was performed using miRNA-specific primers (MSPs). The primer for reverse transcription was designed with double-stranded stem–loop structure with universal reverse primer binding site at 5′ end and last eight nucleotides at the 3′-end complementary to the 3′-end of miRNA. cDNA generated using this Stem-loop-RT primer was used to carried out real time PCR using miRNA-specific forward primers and universal primer with complementarity to the ‘stem’ sequence of the MSP. All primers used are listed in below. Reverse transcription was performed using SuperScript III RT (Invitrogen, California, USA), and the quantitative PCR was carried out using SYBR Green (Applied Biosystems, California, USA) as the fluorescent detector on an Applied Biosystems 7900HT machine. *miR-23a-5p* was analyzed for expression profiles at different conditions as mentioned in the respective experiments and levels where normalized to cellular U6snRNA levels. The primer sequences used were given below.

**Table:** 

Primer Name	Sequence
U6snRNA RT-primer	CTCAACTGGTGTCGTGGAGTCGGCAATTCAGTTGAGAAAAATAT
U6snRNA Forward primer	ACACTCCAGCTGGGGTGCTCGCTTCGGCA
Universal reverse primer	TGGTGTCGTGGAGTCGCAATTCAGTTG
miR23a* RT-primer	CTCAACTGGTGTCGTGGAGTCGGCAATTCAGTTGAGAAATCCCA
miR23a* Forward primer	ACACTCCAGCTGGGGGGGTTCCTGGGGA

### Cloning reporter constructs

Oligos containing three tandem repeat of the complementary sequence to *mir-23a-5p* or TLR-2 3′UTR region were commercially synthesized (Bioserve, Hyderabaed, India), phosphorylated using T4 PNK, annealed, and cloned in to EcoRI and XhoI site in pMXCRGb vector^50^. Positive clones were identified using colony PCR followed by sanger sequencing. Oligo sequences used are:

hsa-miR-23a-5p(forward)-AATTAAATCCCATCCCCAGGAACCCCAAATCCCATCCCCAGGAACCCCAAATCCCATCCCCAGGAACCCC;

hsa-miR-23a-5p(reverse)-5′TCGAGGGGTTCCTGGGGATGGGATTTGGGGTTCCTGGGGATGGGATTTGGGGTTCCTGGGGATGGGATTT3′

TLR2–3′UTR(forward)-5′AATTGACTTCATGGATGCAGAACCCATGACTTCATGGATGCAGAACCCATGACTTCATGGATGCAGAACCCAT3′,

TLR2–3′UTR(reverse)-5′TCGAATGGGTTCTGCATCCATGAAGTCATGGGTTCTGCATCCATGAAGTCATGGGTTCTGCATCCATGAAGTC3′.

TLR2–3′UTR mutant 1 (forward): AATTGACTTCATGGATGCAGATGCCATGACTTCATGGATGCAGATGCCATGACTTCATGGATGCAGATGCCAT

TLR2–3′UTR mutant 1 (reverse): TCGAATGGCATCTGCATCCATGAAGTCATGGCATCTGCATCCATGAAGTCATGGCATCTGCATCCATGAAGTC

TLR2–3′UTR mutant 2 (forward): AATTGACTTCTTGGATGCACATGCCATGACTTCTTGGATGCACATGCCATGACTTCTTGGATGCACATGCCAT

TLR2–3′UTR mutant 2 (reverse): TCGAATGGCATGTGCATCCAAGAAGTCATGGCATGTGCATCCAAGAAGTCATGGCATGTGCATCCAAGAAGTC

### Semi-quantitative real time PCR

Total RNA was extracted using Trizol reagent (Invitrogen, California, USA). Five hundred nanogram of the extracted RNA was converted into cDNA using superscript II cDNA kit (Invitrogen, California, USA). The expression of genes was studied using SYBR green method (Finnzymes F410L, Thermo Scientific, Rockford, IL, USA). The Ct values were normalized with *ACTB* and the fold differences were calculated using 2^−∆∆Ct^ method. The NF-κB array (RT^2^ profiler PCR array human NF-κB signaling target. Qiagen, Hilden, Germany, Catalogue No: PAHS-225z) was performed according to manufacturer’s instructions.

### Immunoblots

NB4 homogenates were obtained by cell lysis in RIPA buffer (Sigma, Missouri, USA), with complete protease inhibitors (Roche, Basel, Switzerland). Nuclear extracts were taken from cells using NE-PER kit (Thermo Scientific, Massachusetts, USA) according to the manufacturer’s protocol. The lysates and elutes were analyzed in SDS–PAGE. After protein transfer to nitrocellulose membrane, membranes were blocked with non-fat dry milk (5%, 2 h) followed by incubation with primary antibodies overnight. The protein bands were detected by standard chemiluminescence method (ThermoScientific, Massachusetts, USA).

### Electroporation

The electroporation of *miR-23a-5p* mimics (Exiqon, Denmark) into NB4 cells were performed using Amaxa electroporator unit (program NB4 X-01) and recovered in complete media for 24 h. Same kit was used to electroporate pMXCRGb and pMXCRGb-TLR2 vectors in NB4 cells. For analysis in vector electroporated experimental set up the cells were gated for RFP positive cells and looked for the expression of GFP upon re-introduction of *miR-23a-5p* mimics.

### Immunofluorescence

The leukemic cells were co-cultured with stromal cells, the cells were carefully pipetted out and cytospin slides were made after 6 h of co-culture. The cells were fixed in 4% paraformaldehyde followed by blocking using 5% goat serum. It was further incubated with primary antibodies such as p65 (Santa Cruz, Dallas, USA) overnight at 4 °C. The slides were rinsed with PBS thrice and incubated with secondary antibodies (anti-mouse) conjugated with alexaflour 594, (Invitrogen, California, USA) for 1 h. The slides were again washed, air dried and counterstained with DAPI containing mountant (Vectashield, California, USA). The images were acquired in fluorescence microscope (Axioimager M1, Carl Zeiss, Germany) at ×100 with oil immersion and images were analyzed using ISIS metasystem, (Metasystems GmbH, Altlussheim, Germany).

### Flow cytometry analysis of TLR2 expression

For the expression of TLR2 (CD282) protein on the leukemic cells, phycoerythrin tagged antibody against TLR2 was purchased (PE Mouse Anti-Human CD282 Clone 11G7) from BD Pharmingen (New Jersey, USA). Briefly the cells were washed and incubated with the antibody in dark for 30 min at 4 °C. The cells were washed to remove unbound antibodies and the re-suspended in PBS-albumin (1%) solution. The cells were acquired in Beckman Coulter Gallios (California, USA) and the data were analyzed in FlowJo software V10.07 (Ashland, Orlando USA).

### Mouse models and drug treatments

FVB/N mice were obtained from Jackson Laboratory (Maine, USA). Mice at 6 to 8 weeks of age were used in all the experiments. The animal study design and euthanasia protocols were approved by the Institutional animal ethics committee (IAEC approval number 17/2012). A well-established transplantable APL mouse model was used in this study^12,51^. Briefly, APL cells from the spleen of MRP8-PML-RAR transgenic mice (FVB/N) were harvested and cryopreserved (a kind gift from Dr. Christine Chomienne with permission from Dr. Scott Kogan). Mouse APL cells (5 × 10^4^ cells/mouse) were injected intravenously via the tail vein into genetically compatible FVB/N recipients, without conditioning with either radiation or chemotherapy. ATO was given as intra-peritoneal at the concentration of 5 mg/kg of mice starting on day 7 post injection of malignant cells and continued for 28 days, while hydroxychloroquine was also given as intra-peritoneal at the concentration of 60 mg/kg of mice starting on day 7 post injection of malignant cells and continued for 28 days. Blood was collected on day 20 by retro-orbital method and the mice were monitored for their survival.

## Supplementary information

Supplementary data

Supplementary table1

Supplementary table 2
