# Supplementary material for: Stromal cells downregulate miR-23a-5p to activate protective autophagy in acute myeloid leukemia
Source: Cell Death Dis. 2019 Sep 30;10(10):736. doi: 10.1038/s41419-019-1964-8 (PMC6769009; doi:10.1038/s41419-019-1964-8)

**Stromal-cells down-regulate m*iR-23a-5p* to activate protective autophagy in acute myeloid leukemia**

Saravanan Ganesan^1*^, Hamenth Kumar Palani^1*^, Vairavan Lakshmanan^2^, Nithya

Balasundaram^1^, Ansu Abu Alex^1^, Sachin David^1^, Arvind Venkatraman^1^, Anu Korula^1^, Biju George^1^, Poonkuzhali Balasubramanian^1^, Dasaradhi Palakodeti^2^, Neha Vyas^3#^, Vikram Mathews^1#^.

1. Department of Haematology, Christian Medical College, Vellore, India.
2. Institute for Stem Cell Biology and Regenerative Medicine (InStem), Bengaluru, India.
3. Molecular Medicine Department, St. John's Research Institute, St. John's National Academy of Health Sciences, Bengaluru, India.

*- Equal Contribution

**Supplementary file**

**Supplementary Results**

**Supplementary figure 1:**

Stromal cells protect the malignant promyelocytes from ATO induced apoptosis both in contact dependent and independent system as showed by reduced cleavage of pro-caspase3 in leukemic cells treated with ATO in presence of stroma (direct culture and transwell culture) (n=3).


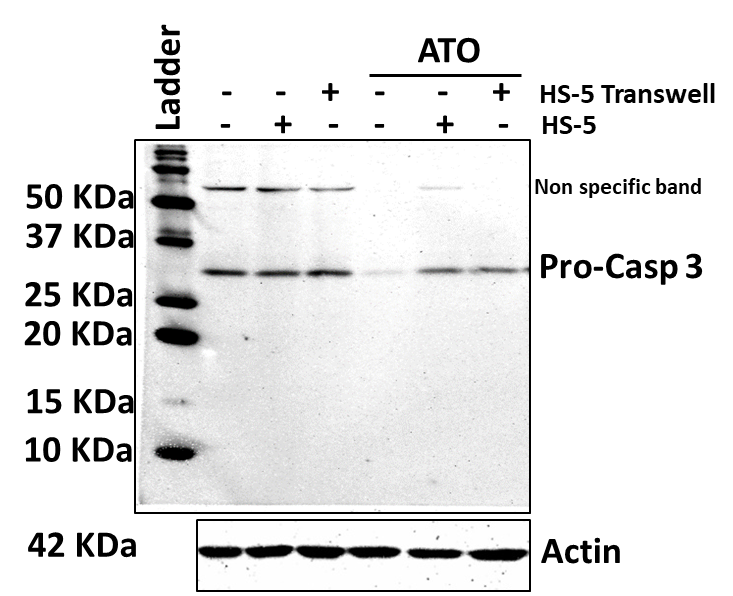


**Supplementary figure 2:**

Volcano plot after adjusted pvalues showing significant down-regulation of *miR-23a-5p* alone in the entire analysis.


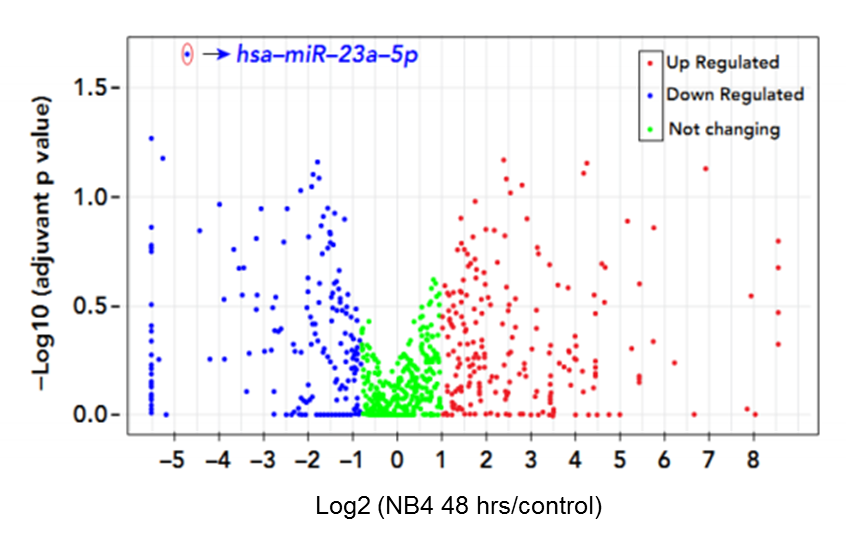


**Supplementary figure 3:**

Activation of NF-kB pathways in leukemic cells (NB4) upon co-culture with stromal cells, the image shows an increased translocation of p65 subunit in the nucleus of NB4 cells upon co-culture with stromal cells (n=3) at the magnification of 100x.


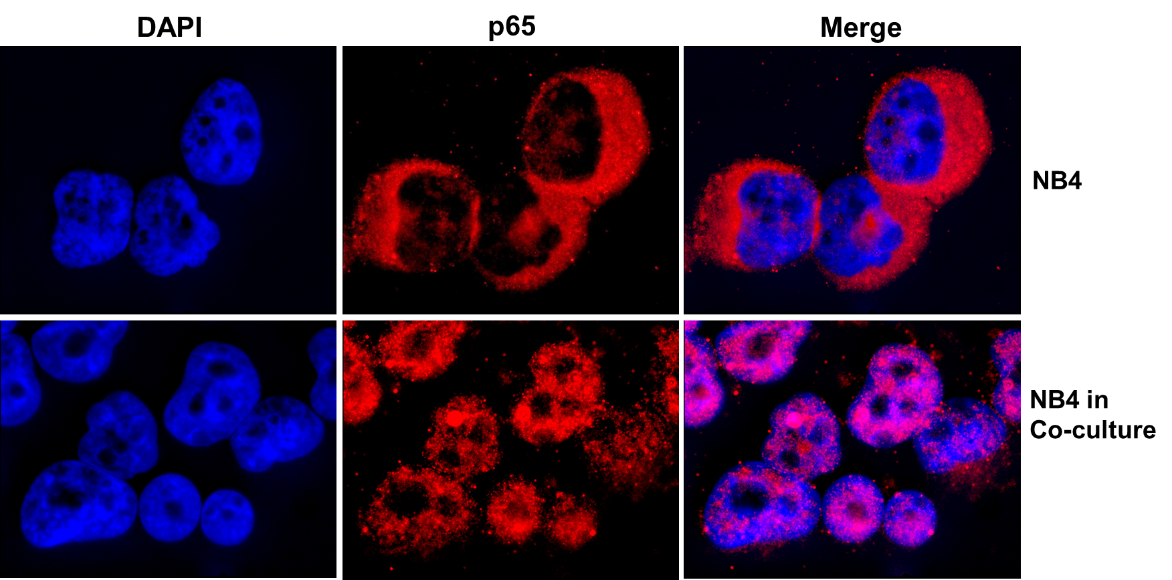


**Supplementary figure 4:**

NF-kB target genes RT-PCR array showing up-regulation of NF-kB targets in NB4 cells upon co-cultured with HS-5 cells even in the presence of ATO compared to NB4 alone control at 48 hours (n=3). The expression of control is denoted as a line at 1 in the Y axis.


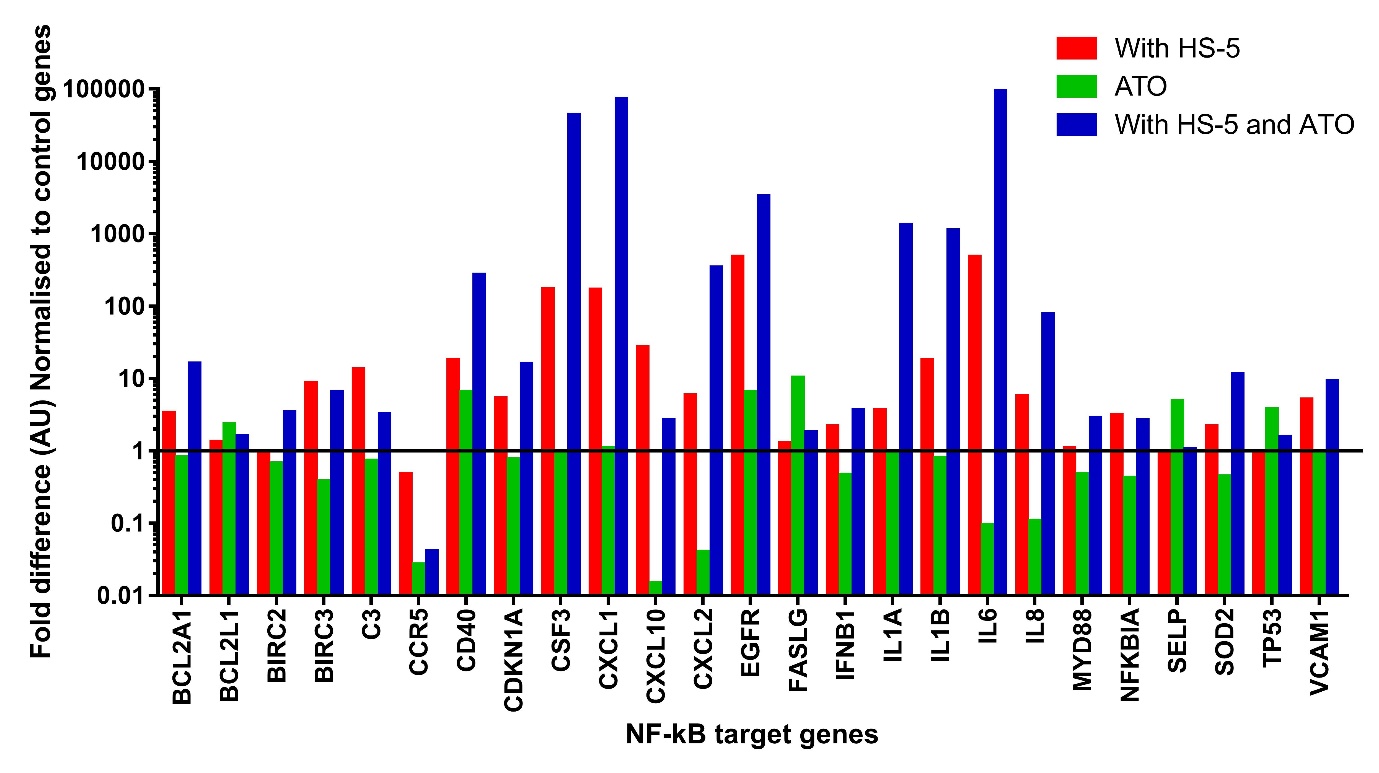


**Supplementary figure 5:**

Inability of NB4/GFP-MAD cells to activate NF-kB pathway demonstrated by a) decreased or no translocation of p65 in the nucleus of the cells even in the presence of stroma (n=3). The image was captured at the magnification of 100x. b) Reduced expression of NF-kB target genes in NB4/GFP-MAD cells compared to NB4 alone (n=3) evaluated by a real time PCR assay.

a)


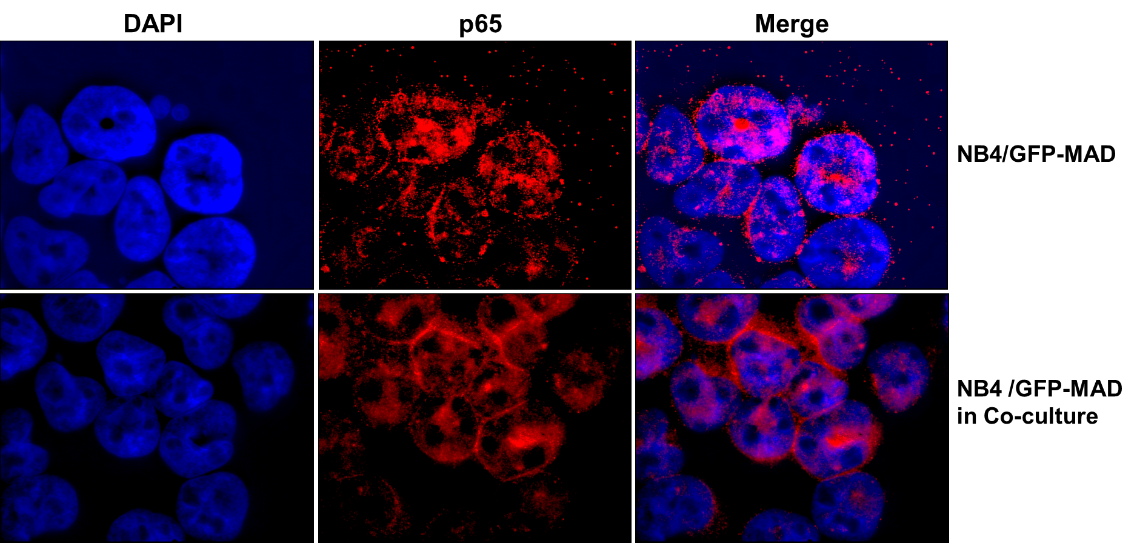


b)


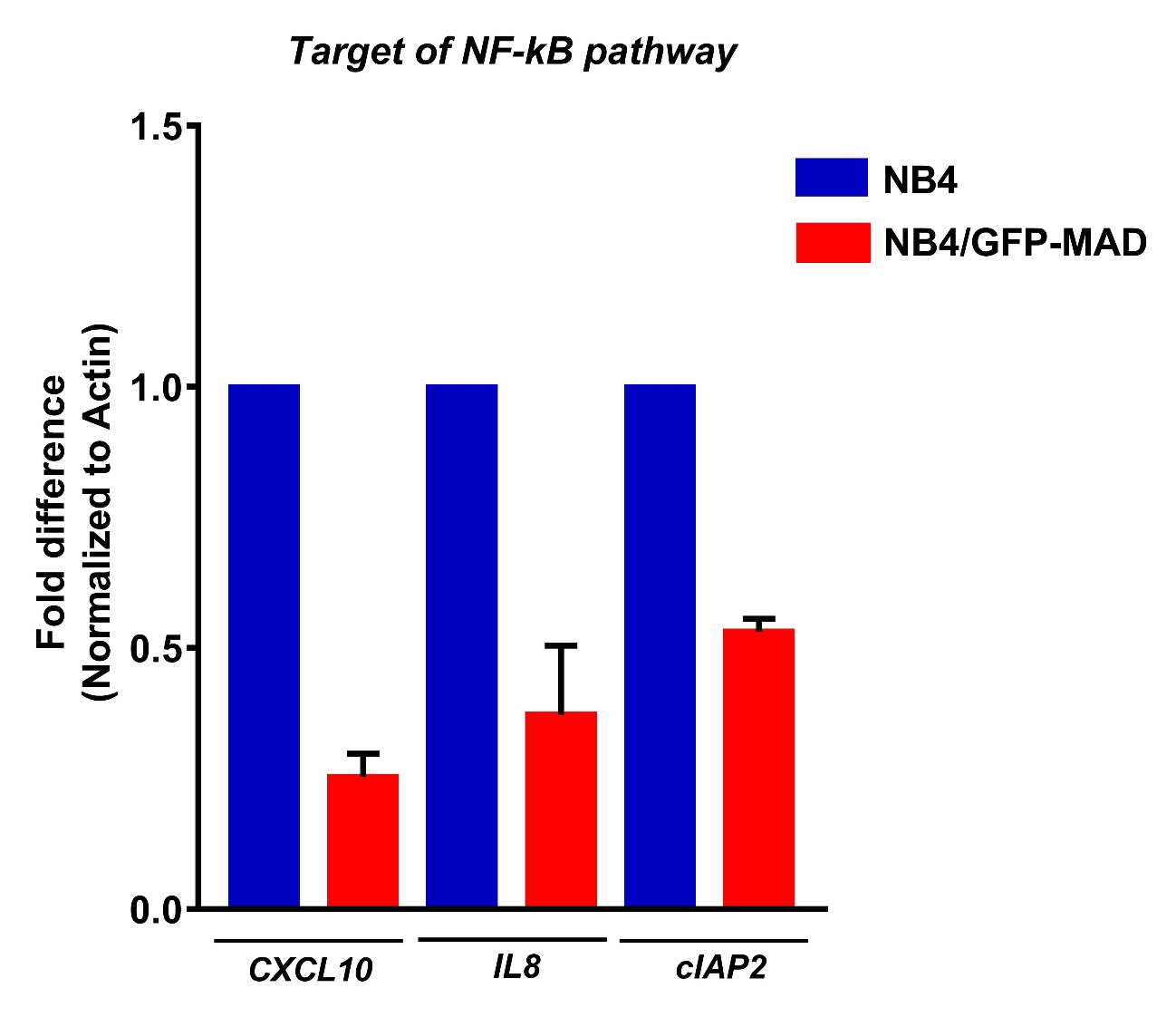


**Supplementary figure 6:**

GFP-miR-23a-5p- reporter (pMXCRGb vector) showing reduced intensity of GFP upon re-introduction of *miR-23a-5p* mimic in NB4 cells compared to controls (n=3).


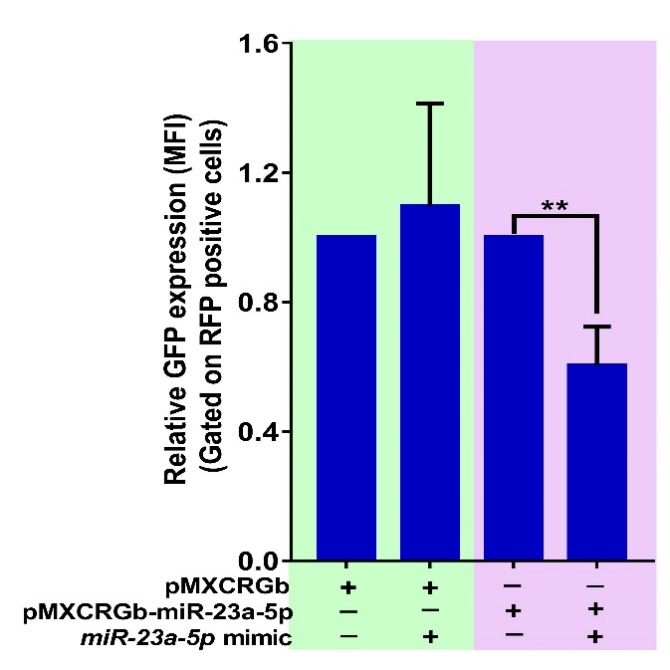


**Supplementary figure 7:**

Overexpression of miR-23a-5p in leukemic cells overcomes the protective effect mediated by stromal cells as evidenced through reduction in the pro caspase-3 levels (n=3).


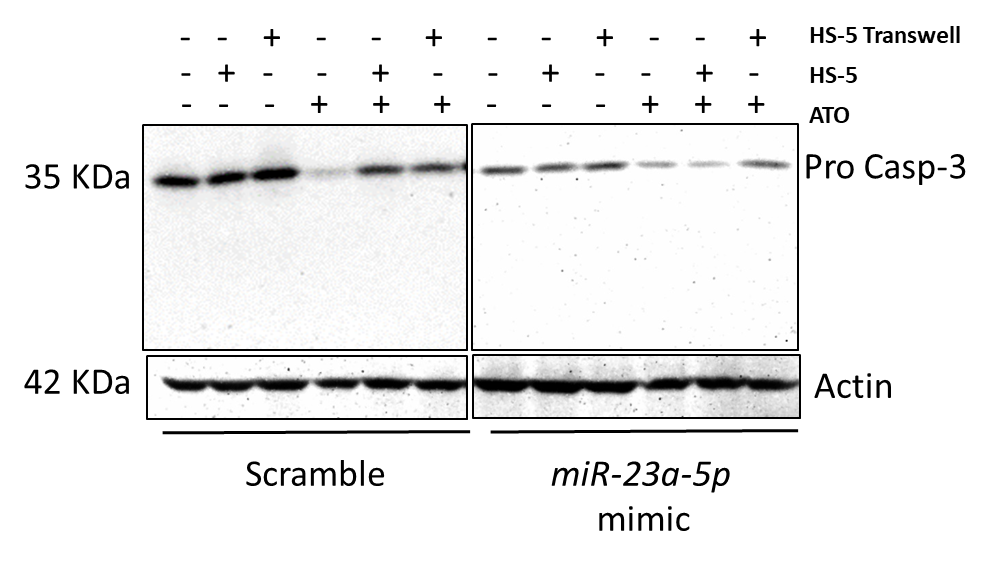


**Supplementary figure 8:**

Re-introduction of *miR-23a-5p* mimic into NB4 cells restores sensitivity against DNR in the presence of stroma (n=3), viability was measured using Annexin V/7AAD kit. NB4-GFP/MAD cells having high expression of miR-23a-5p did not show a protective effect against DNR in the presence of stroma (n=3). **-P= 0.005, *-P=0.02, NS- Not significant.


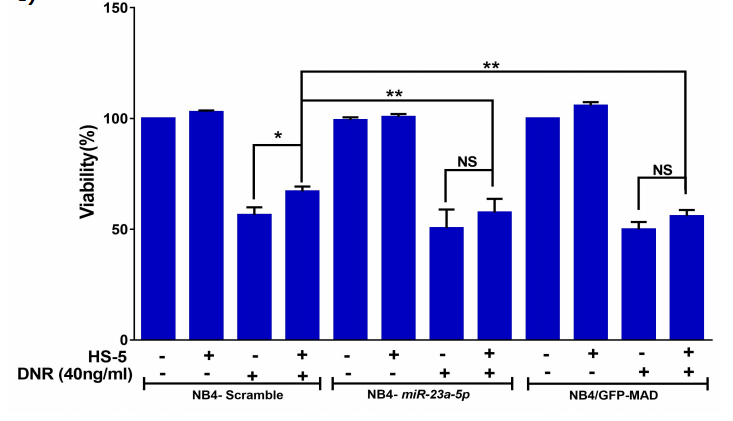


**Supplementary figure 9:**

Expression of *TLR2* (transcript) in leukemic cells upon co-culture and in the presence of *miR-23a-5p* mimic (n=3).


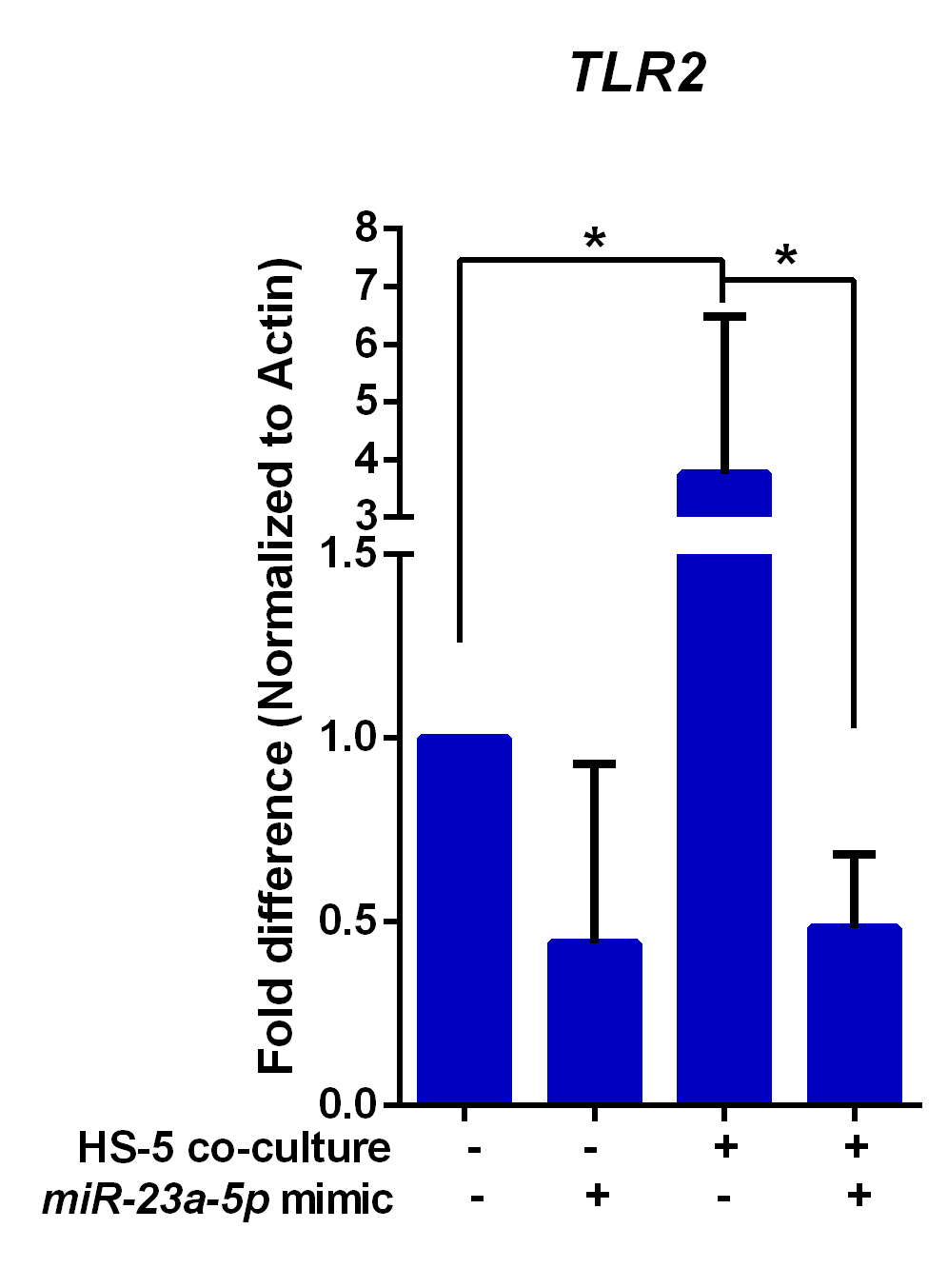


**Supplementary figure 10:**

Effect of co-culture and miR-23a-5p mimic on autophagy genes: Autophagy genes at the transcript levels were not affected by co-culture and in the presence of miR-23a-5p (except *ATG9B*) in NB4 cells upon co-culture.


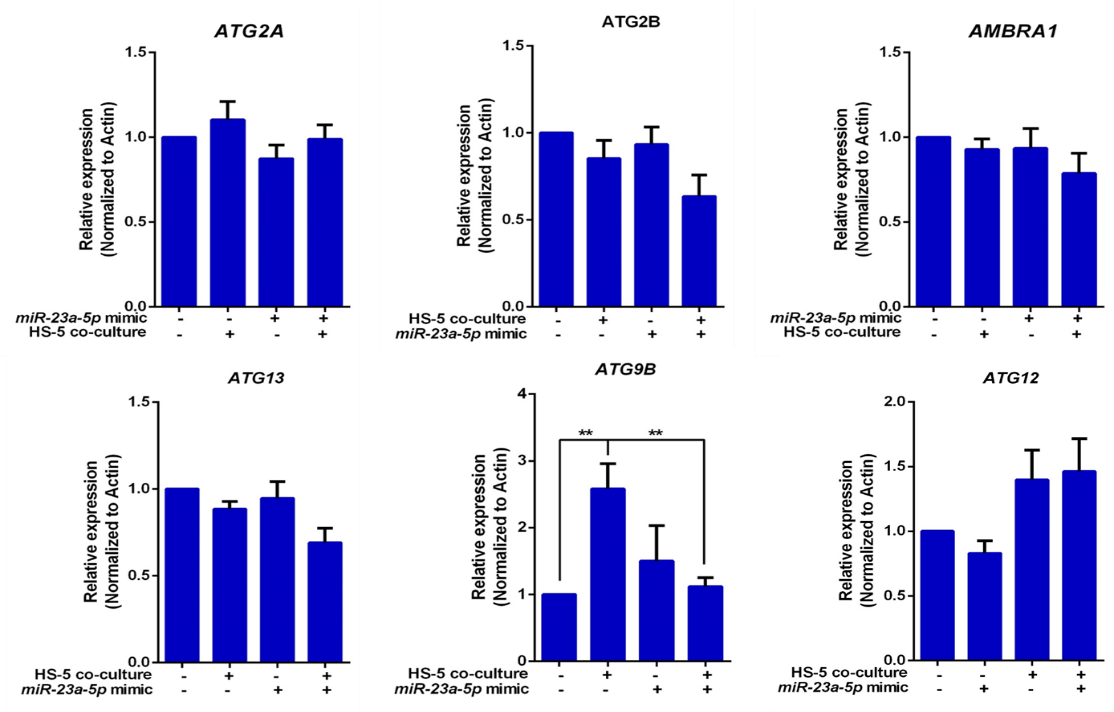


**Supplementary figure 11:**

Expression of *TLR2* transcript (a) and protein (b) in NB4/GFP-MAD cells compared to NB4 cells alone (n=3).

**
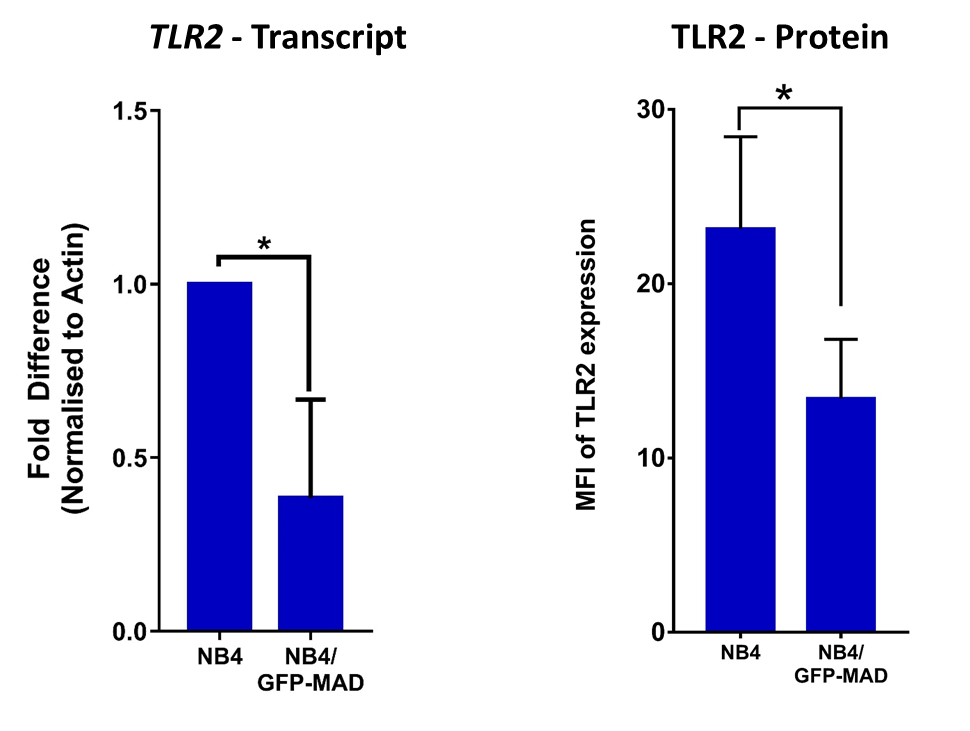
**

**Supplementary figure 12:**

Knockdown of *TLR2* in leukemic cells upon followed by co—culture with stromal cells did not show an increase in autophagy gene (n=3). Co-culture was done was 48 hours before collecting proteins.


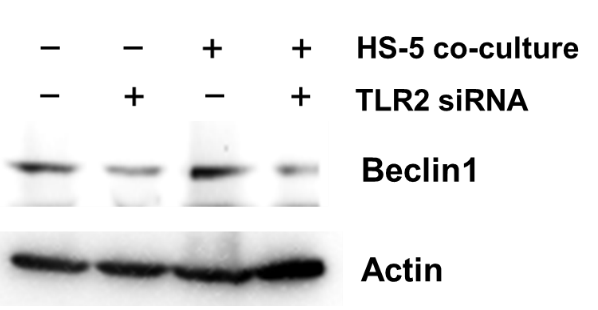


**Supplementary figure 13:**

Knock down of TLR2 in NB4 cells using TLR2 siRNA, the assay was performed using flow cytometry post 24 hours electroporation of siRNA. Shown below is the representative histogram of TLR2 knock down in NB4 cells (n=3).


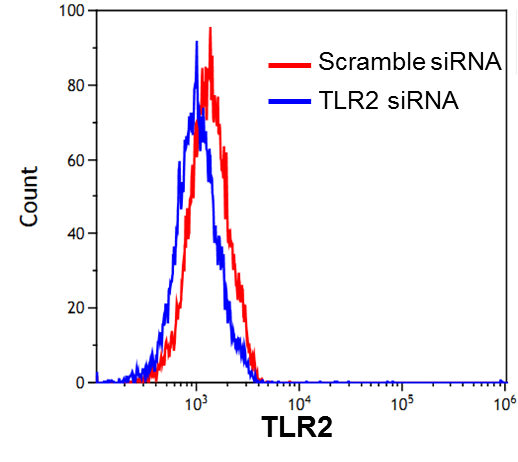


**Supplementary figure 14:**

Stromal cells induces a protective effect against arsenic trioxide in AML cell line a) THP1 ,b) Kasumi-1 and c) primary AML cells (n=10) in both contact dependent and independent systems (n=3). Viability was assessed using Annexin V /AAD kit, post 48 hours treatment with daunorubicin (DNR-40ng/ml) and cytarabine (Ara-C- 400ng/ml). The viability of untreated cells were normalized to 100 % and the treated cells viability were compared to normalized untreated cells.


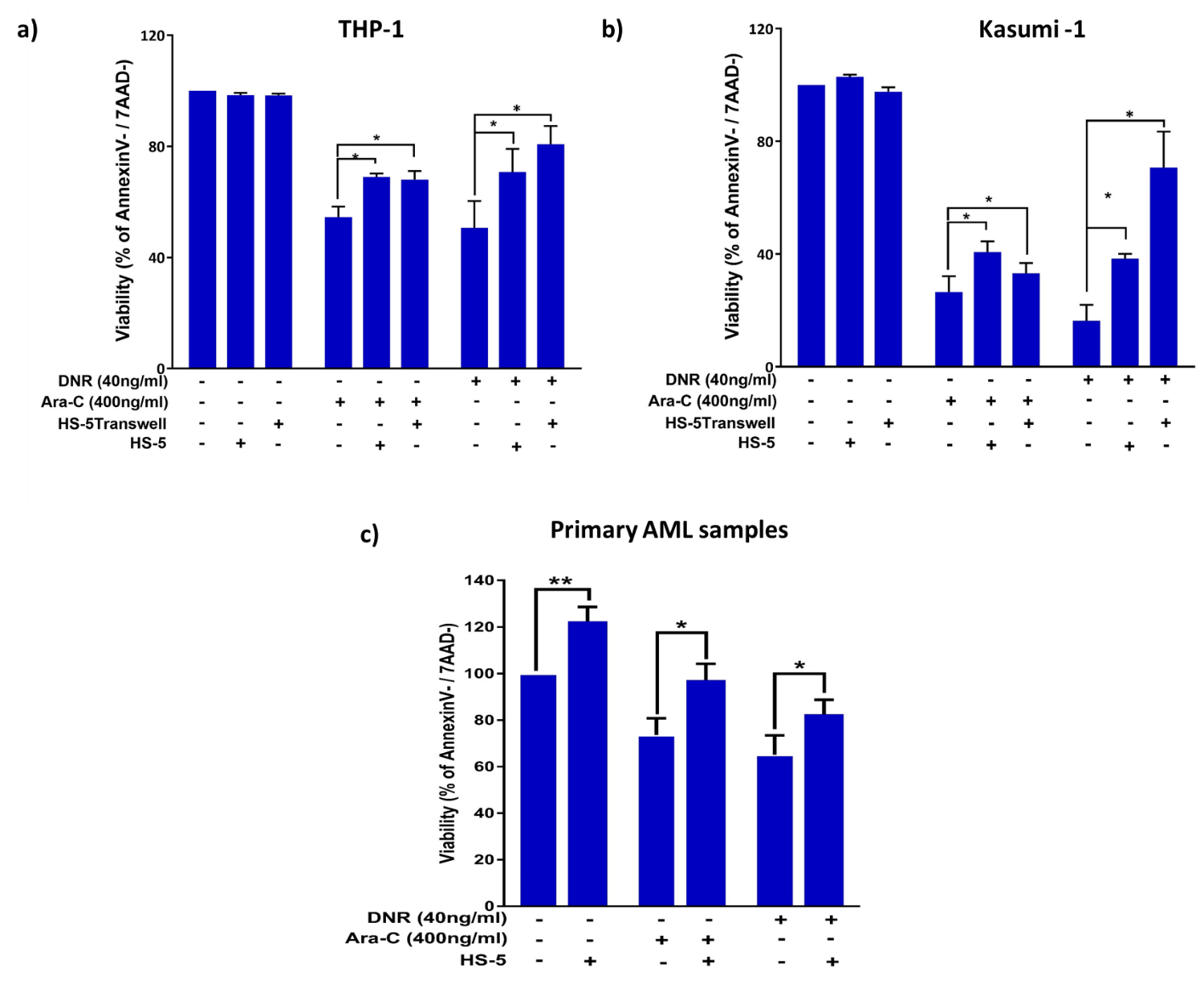


**Supplementary figure 15:**

Activation of NF-kB signaling in AML cells (U937) upon co-culture with stromal cells; a) Immunofluorescence showing increased translocation of p65 subunit in the nucleus of U937 when co-cultured with stroma (n=3); The image was captured at the magnification of 100x. b) Increased expression of NF-kB target genes in U937 cells when co-cultured with stromal cells analyzed by Q-PCR.


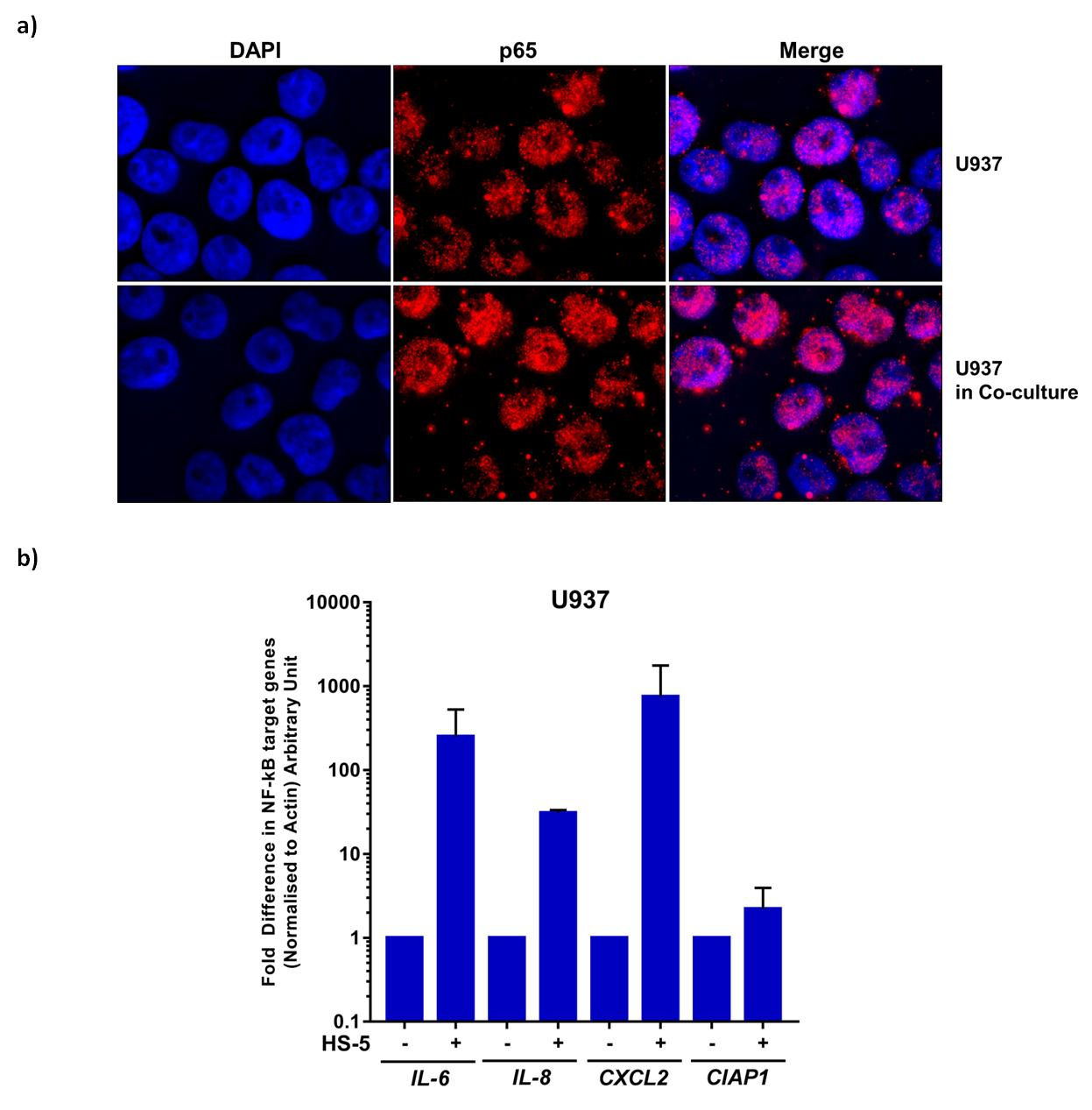


**Supplementary figure 16:**

Basal expression of miR-23a-5p in different leukemic cell lines, compared to NB4 cells; NB4/GFP-MAD and Kasumi-1 cell lines had higher expression of *miR-23a-5p* while late myeloid differentiated leukemic cells such as U937, HEL and THP1 cell lines had lower expression of *miR-23a-5p* (n= atleast 3 per cell line).


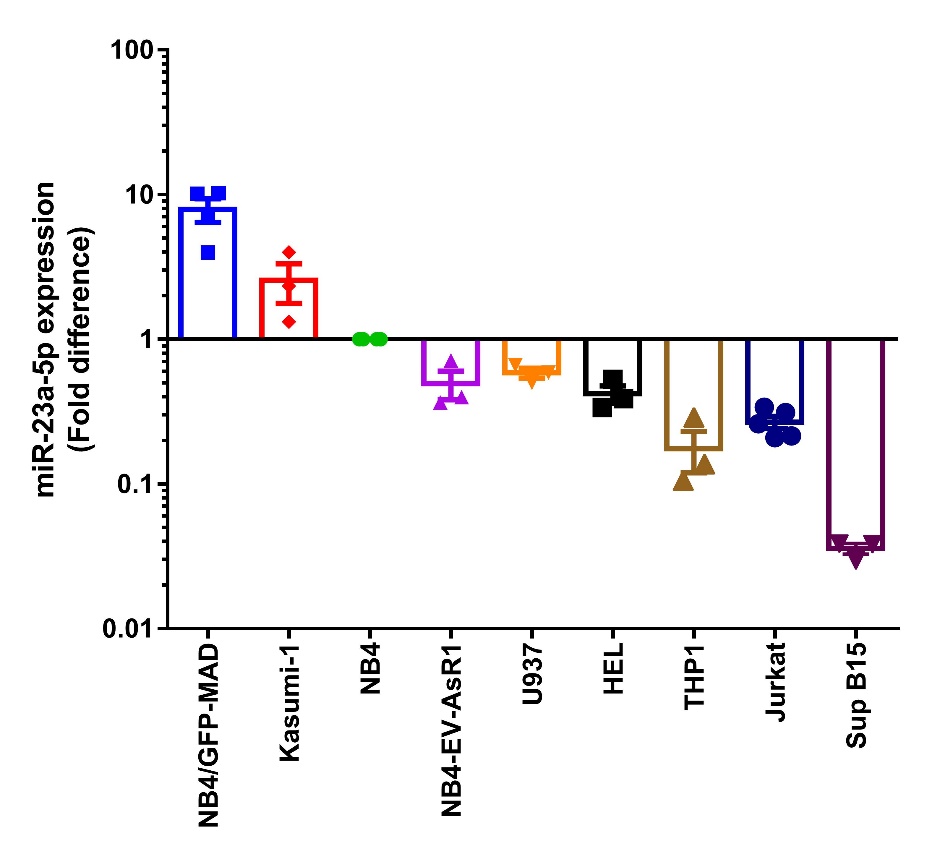


**Supplementary figure 17:**

Daunorubicin increased the expression of miR-23a-5p in U937 cells (n=3) as detected through real time PCR assay.

**
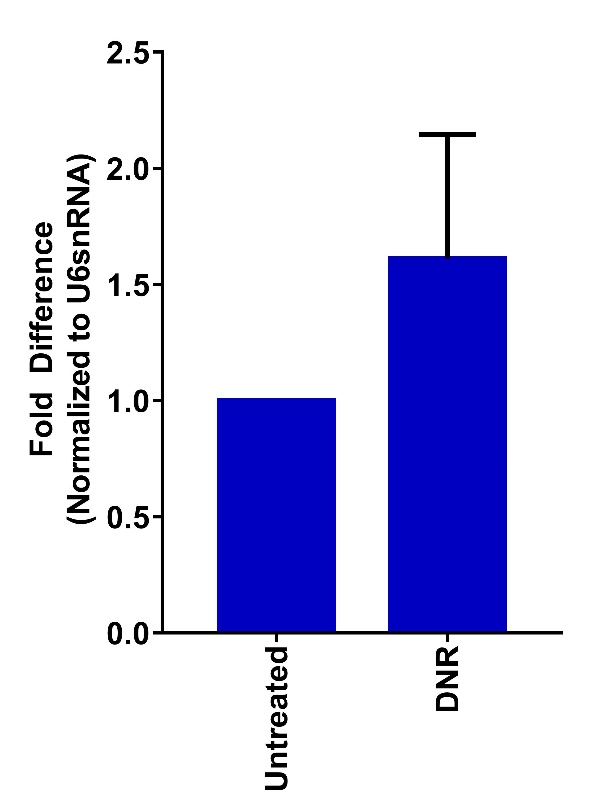
**

**Supplementary figure 18:**

Re-introduction of *miR-23a-5p* mimic into THP-1 cells does not restore sensitivity against DNR in the presence of stroma (n=3), viability was measured using Annexin V/7AAD kit.


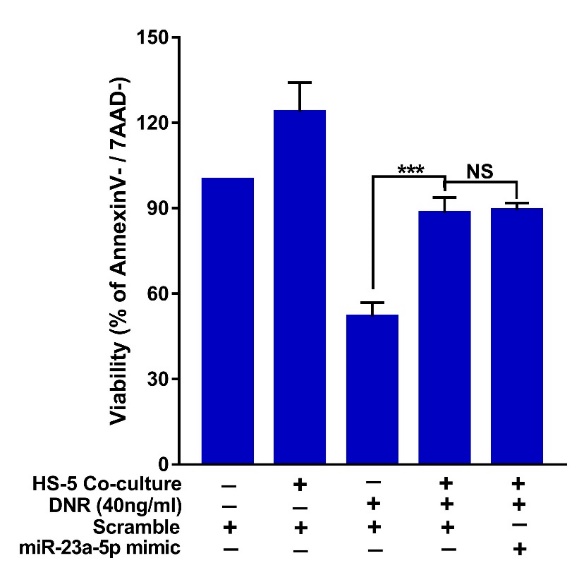


**Supplementary figure 19:**

Expression of *TLR2* (2- ΔΔCt - fold difference) in U937 and THP1 cells co-cultured with stromal cells (HS-5) (n=3).


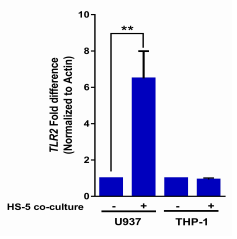


**Supplementary figure 20:**

Treating NB4 cells with autophagy inhibitors bafilomycin A1 (BAFA1- 10nM) and hydroxyl chloroquine (HCQ- 10uM) resulted in the inhibition of autophagy as observed in accumulation of p62 and LC3 bands (n=3).

**
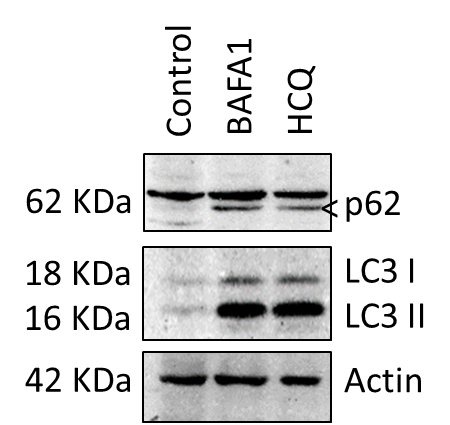
**

**Supplementary figure 21:**

Inhibition of autophagy using autophagy inhibitors (bafilomycin A1- 10nM and hydroxychloroquine -10uM) restores the sensitivity of ATO (2uM) in in primary APL cells; n=3) even in the presence of stroma, assays were done at the end of 48 hours using apoptosis assay. (*- P=0.02, **-P=0.005).


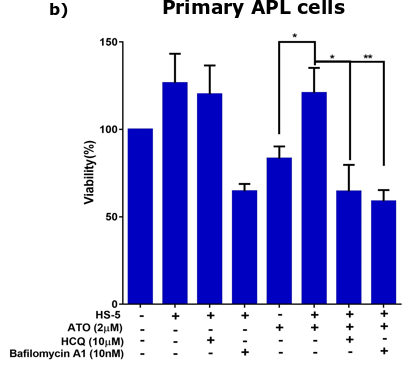


**Supplementary figure 22:**

Inhibition of autophagy using autophagy inhibitors (bafilomycin A1- 10nM and hydroxychloroquine -10uM) restores the sensitivity of DNR (40ng/ml) in primary AML cells (n=3) in the presence of stroma, assays were done at the end of 48 hours using apoptosis assay (AnnexinV /7AAD kit).


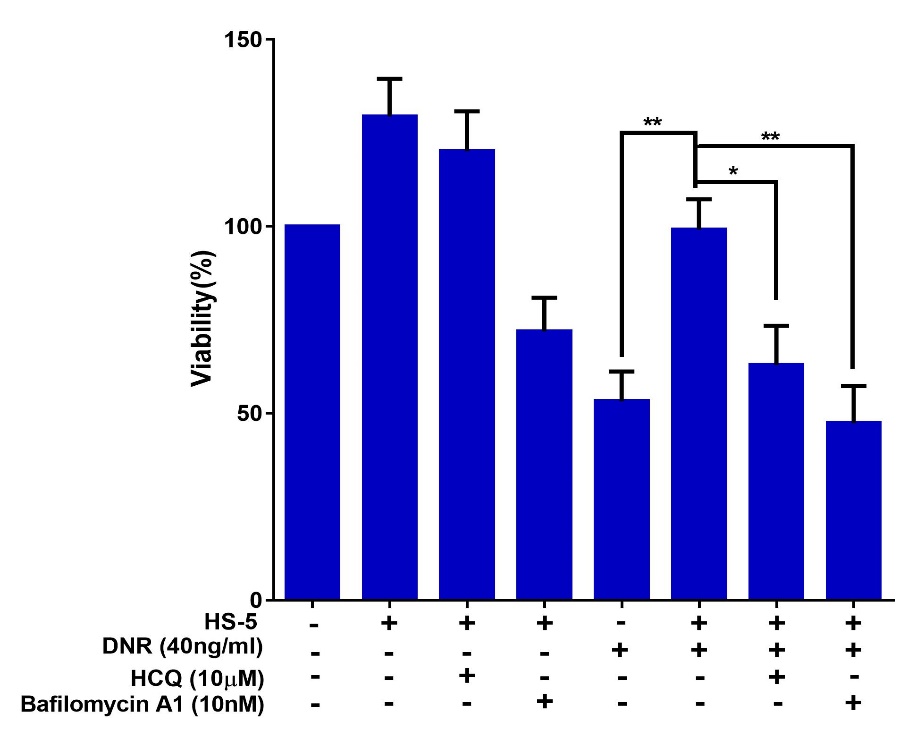


**Supplementary figure 23:**

HCQ when combined with ATO, reduced the tumour burden in the APL mice evidenced through reduced *PML-RARA* copy number in the peripheral blood of APL mice on day 20 compared to placebo, ATO or HCQ alone arm


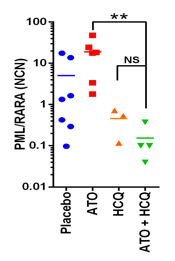


**Supplementary figure 24:**

Nuclear accumulation of B-Catenin in NB4 cells upon co-culture with stromal cells and treated with drugs such as arsenic trioxide (ATO -2uM) and bortezomib (BTZ – 200nM) for 24 hours (n=3).


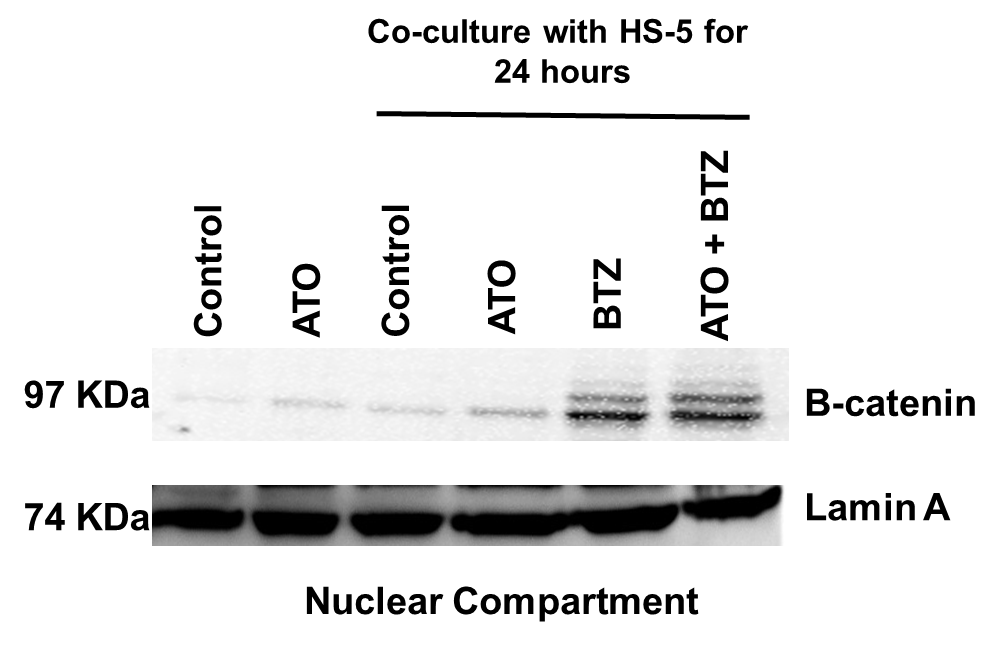

Supplement: Supplementary file 1 — Supplementary data [file 41419_2019_1964_MOESM1_ESM.doc]
